# A comparative study of three bone age assessment methods on Chinese preschool-aged children

**DOI:** 10.3389/fped.2022.976565

**Published:** 2022-08-16

**Authors:** Chengcheng Gao, Qi Qian, Yangsheng Li, Xiaowei Xing, Xiao He, Min Lin, Zhongxiang Ding

**Affiliations:** ^1^Department of Radiology, Affiliated Hangzhou First People’s Hospital, Zhejiang University School of Medicine, Hangzhou, China; ^2^Department of Radiology, The Third Affiliated Hospital of Zhejiang Chinese Medical University, Hangzhou, China; ^3^Center for Rehabilitation Medicine, Department of Radiology, Zhejiang Provincial People’s Hospital, Affiliated People’s Hospital, Hangzhou Medical College, Hangzhou, China; ^4^Key Laboratory of Clinical Cancer Pharmacology and Toxicology Research of Zhejiang Province, Hangzhou, China

**Keywords:** bone age assessment, preschool children, Greulich–Pyle (GP), Tanner–Whitehouse 3 (TW3), China 05 RUS–CHN

## Abstract

**Background:**

Bone age assessment (BAA) is an essential tool utilized in outpatient pediatric clinics. Three major BAA methods, Greulich–Pyle (GP), Tanner–Whitehouse 3 (TW3), and China 05 RUS–CHN (RUS–CHN), were applied to comprehensively compare bone age (BA) and chronological age (CA) in a Chinese sample of preschool children. This study was designed to determine the most reliable method.

**Methods:**

The BAA sample consisted of 207 females and 183 males aged 3–6 years from the Zhejiang Province in China. The radiographs were estimated according to the GP, TW3, and RUS–CHN methods by two pediatric radiologists. The data was analyzed statistically using boxplots, the Wilcoxon rank test, and Student’s *t*-test to explore the difference (D) between BA and CA.

**Results:**

According to the distributions of D, the boxplots showed that the median D of the TW3 method was close to zero for both male and female subjects. The TW3 and RUS–CHN methods overestimated the age of both genders. The TW3 method had the highest correct classification rate for males but a similar rate for females. The GP method did not show any significant difference between the BA and CA when applied to 3-year-old males and 4-year-old females while the TW3 method showed similar results when applied to 6-year-old females. The RUS–CHN method showed the least consistent results among the three methods.

**Conclusion:**

The TW3 method was superior to the GP and RUS–CHN methods but not reliable on its own. It should be noted that a precise age diagnosis for preschool children cannot be easily made if only one of the methods is utilized. Therefore, it is advantageous to combine multiple methods when assessing bone age.

## Introduction

The bone age assessment (BAA) is a commonly used procedure in pediatric clinics, including subspecialties such as Endocrinology and Orthopedics (1–3). Bone age (BA) is the primary indicator of maturity status in a child, as various diseases affecting growth can result in a significant discrepancy between bone age and chronological age (CA) (3–6). Therefore, choosing a reliable BAA method for clinic use is essential.

Three major BAA methods are currently applied in Chinese pediatric practices: the Greulich–Pyle (GP) method (7), the Tanner–Whitehouse 3 (TW3) method (8), and the China 05 RUS–CHN (RUS–CHN) method (9). All three methods can be used to assess the development during early and middle childhood, both of which are the stages of rapid growth.

Recently, the performance of a specific BAA method applied in populations from different age groups has been analyzed and reported extensively (2, 10, 11). However, only a few comprehensive studies have used three different BAA methods to correlate results among preschool children. The study aimed to compare the performance of the GP, TW3, and RUS–CHN methods for BAA in a subgroup of Chinese preschool children.

## Materials and methods

### Data acquisition

**FIGURE 1:**
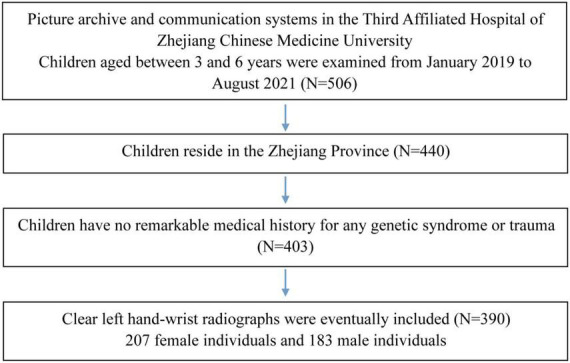
Flowchart of sample selection process.

We selected 390 left hand-wrist radiographs from the Third Affiliated Hospital of Zhejiang Chinese Medicine University (Hangzhou, China) from January 2019 to August 2021. Radiographs were obtained from the picture archive and communication systems. The subjects resided in the Zhejiang Province and had no remarkable medical history of genetic syndrome or trauma. A total of 390 subjects, consisting of 207 females and 183 males aged 3–6 years, were included in this study (Figure 1).

Each radiograph for BAA could be evaluated using the following three methods: (i) the GP method comparing a hand-wrist radiograph of a child with the standard films applied in the age-matched atlas, published in 1959 (7); (ii) the TW3 method scoring the skeletal maturity for each hand and wrist bone, published in 2001 (8); (iii) the RUS–CHN method analyzing the skeletal development standards of hand and wrist for children in China, version 05–I, published in 2006 (12).

The skeletal age was analyzed independently for each of the radiographs based on the different assessment methods described above. In order to assess the interobserver error, two pediatric radiologists with extensive training and experience in BAA assessed the radiographs. The differences in the results were not significant, and the average value was considered as the BA.

To compare the difference in performance between the three methods applied in this study, the difference (D) between the CA and BA was calculated (D = CA - BA). Specifically, D represented the underestimation (positive value) or overestimation (negative value).

The Ds of underestimation, overestimation, and correct classifications were determined separately, based on the classification of age measured by CA ± 3 months. Therefore, children whose predicted BA was within CA ± 3 months were considered to be classified appropriately. The two main reasons for assuming this span of age are as follows: (i) the age span predicted using the GP method is in intervals of approximately 6 months (e.g., 3 years, 3 years and 6 months, 4 years and 2 months) (7); (ii) age ± 3 months is regarded as the smallest range for BAA, as assessed by left hand-wrist X-rays (13).

In China, children are enrolled in school at the age of 7 years (14, 15), which is the threshold for BAA. Thus, three different methods have been studied in the sample of children aged 3.01–year–old to 6.99–year–old.

### Data analysis

Data was analyzed using the SPSS statistical software (version 25.0, SPSS, Inc., Chicago, IL, United States). Paired Student’s *t*-test was used to compare CA with BA computed by the GP, TW3, and RUS–CHN methods for the cohort stratified by gender at each age point. Frequency tests were carried out to analyze the correlation between CA and the discrepancy between CA and BA for at least 3 months. Then, two tests of the hypothesis were used to assess the correlation between CA and BA. A *p*-value < 0.05 was deemed significant in all tests.

## Results

### Accuracy of the three methods

**FIGURE 2:**
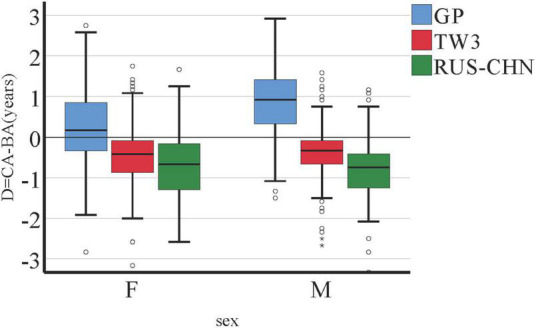
Boxplots of D = CA (chronological age) - BA (bone age), by sex.

In the boxplots, the distributions of the D between CA and BA were represented compactly. According to Figure 2, the median D of the TW3 method is close to zero for male (−0.39) and female (−0.40) subjects. In addition, the variability of TW3 in the boxplots was smaller than that of GP and RUS–CHN.

**FIGURE 3:**
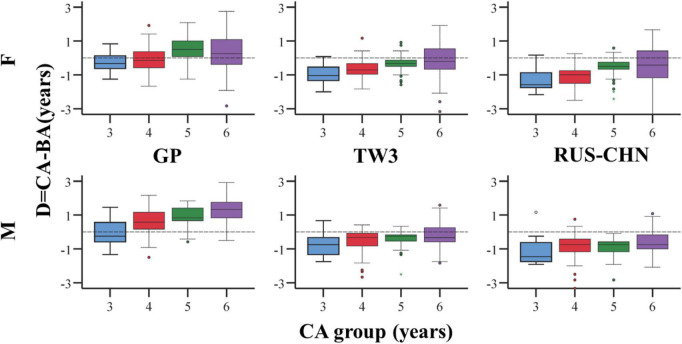
Boxplots of D = CA - BA reported for females (top) and males (bottom) for the three methods and each class of age.

In Figure 3, boxplots were separated by age (3–6 years). Evidently, TW3 and RUS–CHN overestimated the age of both male and female subjects, regardless of age. On the other hand, the D in GP might be affected by the age of the individuals (especially in males), with an escalating trend from the age of 3 to 6 years (positive values). In GP method, D was more than zero in 4-, 5-, 6-year-old male group and 5-, 6-year-old female group, which means BA was underestimated. And in 3-year-old male group and 3-, 4-year-old female group, BA was overestimated.

### Age prediction

The subsets of subjects with underestimated (under), correctly classified (CC), and overestimated (over) CA regarding CA ± 3 months are summarized in Table 1. Subsequently, the accuracy of these methods in CA prediction was determined.

**TABLE 1:** Percentages of subjects with correctly classified (CC), underestimated (under), and overestimated (over) age with respect to the class of CA ± 3 months.

	GP		TW3		RUS–CHN	
	Males	Females	Males	Females	Males	Females
Under (%)	139 (75.96)	95 (45.90)	24 (13.11)	35 (16.91)	12 (6.56)	25 (12.08)
CC (%)	22 (12.02)	53 (25.60)	59 (32.24)	50 (24.15)	22 (12.02)	43 (20.77)
Over (%)	22 (12.02)	59 (28.50)	100 (54.65)	122 (58.94)	149 (81.42)	139 (67.15)

When the D is < 3 months between the CA and BA, indicating that BA is within the range of CA ± 90 days, the correct classification of age was achieved in this study. For males (Table 1), the highest correct classification was achieved using the TW3 method, while for female individuals, the rates of CC in all three methods were similar. Also, it was confirmed that the TW3 and RUS–CHN methods overestimated the age of the subjects (Table 1). Although the percentage of the correct classification in the TW3 method was significantly higher than in the other two methods, it was not a completely reliable result because the accuracy was less than 50%, and that 3-month span was a limited parameter.

### Hypothetical test

To further confirm the reliability of the three different BAA methods, we conducted a statistical reasoning process of the mean values of CA and BA. This would determine whether the values varied by the whole sample based on gender (divided by male and female gender) and in an age-dependent manner.

Two types of hypothesis tests were used to compare CA and BA estimated by GP, TW3, and RUS–CHN methods: (i) Wilcoxon signed-rank test was used to evaluate whether the average rank of CA is different from BA. (ii) Student’s *t*-test was applied to evaluate the significant D in the means of CA and BA.

For both tests, a null hypothesis stated that the two sets (CA and BA) have equal mean ranks. Notably, one difference exists between these two tests: the former is a non-parametric test which does not assume that the population data should be a normal distribution, whereas the latter is a parametric test that assumes the opposite.

**FIGURE 4:**
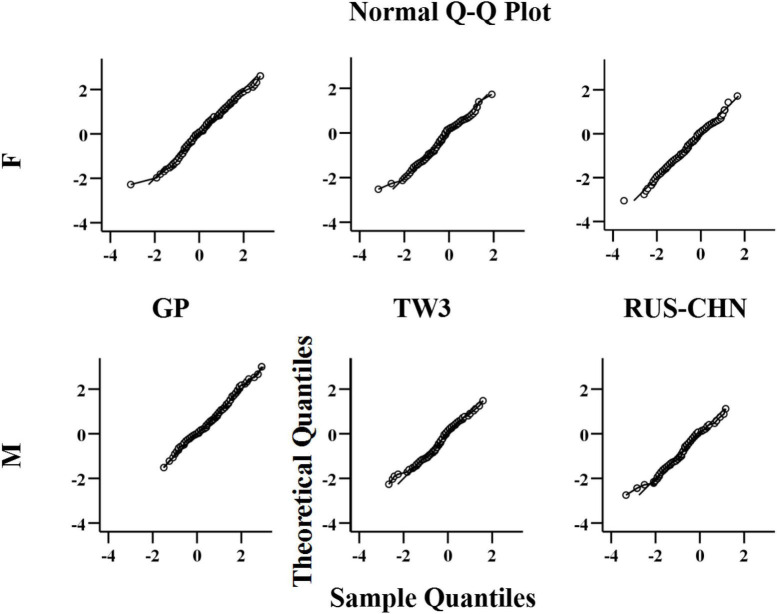
Q-Q plots for D, by sex, for females (top) and males (bottom).

In the Q-Q plots for all three BAA methods (Figure 4), the distribution of the difference (D) is close to the normal distribution. This partially proves that the Student’s *t*-test in this sample is optimal. The Wilcoxon signed-rank test can be adopted.

The present study aimed to understand whether any BAA method can evaluate the actual age; it is an optimal indicator when the *p*-value is > 0.05. The analysis of the age groups revealed similarities between the two test results among the three BAA methods applied to the sample of 183 males and 207 females (*p* < 0.05). This phenomenon clarified that the Wilcoxon test only accepted the null hypothesis for the 3-year-old male and 4-year-old female groups using the GP method and the 6-year-old female group using the TW3 method. In comparison, the RUS–CHN method was the most unreliable because the tests rejected all the null hypotheses (Table 2).

**TABLE 2:** *P*-values for the performed hypothesis tests by sex and age group.

Age group	n.M and n.F	Wilcoxon sign rank test			Student’s *t*-test		
		GP	TW3	RUS–CHN	GP	TW3	RUS–CHN
3	24	0.627	<0.001	<0.001	0.684	<0.001	<0.001
	24	0.041	<0.001	<0.001	0.043	<0.001	<0.001
4	46	<0.001	<0.001	<0.001	<0.001	<0.001	<0.001
	36	0.441	<0.001	<0.001	0.668	<0.001	<0.001
5	39	<0.001	<0.001	<0.001	<0.001	<0.001	<0.001
	67	<0.001	<0.001	<0.001	<0.001	<0.001	<0.001
6	74	<0.001	0.034	<0.001	<0.001	0.049	<0.001
	80	0.007	0.260	0.002	0.004	0.150	0.001
Total	183	<0.001	<0.001	<0.001	<0.001	<0.001	<0.001
	207	<0.001	<0.001	<0.001	<0.001	<0.001	<0.001

For each group, the size is given (n.M and n.F).

## Discussion

Bone age assessment is commonly and widely used in the clinic, reflecting the skeletal maturity, evaluating the growth status, and predicting the future height of children. Although there is an increasing demand for the accurate age estimation of children, studies comparing the reliability of different BAA methods are yet lacking. In China, there are currently a large number of preschool children in the stages of rapid development (ages 3–7). Thus, BAA is of high clinical utility in this population (1, 5, 13, 16–18).

In recent years, studies have reported the inaccuracies of BA predicted by several methods, especially for those under the CA of 7 years (6, 19). The GP method was developed using data from American children in Ohio in the 1950s who belonged to reasonably comfortable families with no medical conditions. This method is simple and easy to implement to determine the BA from 0 to 18 years (7). The original sample for the TW3 method was obtained from a group of healthy British children (8). The TW3 method is time-consuming and complex and thus might require four times longer duration than the GP method. The first sample group consisted of healthy children from upper-middle-class families in the late 1980s (9). The RUS–CHN method was re-assigned for wrist bone morphology according to the classification of the TW series. The different functions and results of the three methods are discussed separately.

### Greulich–Pyle method

The GP method is a procedure in which BA can be assessed by evaluating the subject’s X-ray film with the closest reference in the atlas (7). It is a low-cost and widely available method aimed at a specific group of people. The 57 reference images (31 from males and 26 from females) originated from a sample obtained half a century ago. This procedure has a disadvantage that there are huge differences between the reference standards in the series of pictures collected at intervals of 6 months.

Several studies have applied the GP method in clinical and forensic fields to find that the reliability is not consistent. The GP atlas cannot be applied to all populations (20). Age and gender differences were obvious. Suri et al. (10) found the GP method to be reliable in females and accurate in the 4-year-old age group. Other studies (19, 20) demonstrated that the GP method is likely to underestimate the age in males and females, especially in 3-, 4-, and 6-year-old male group. A large error was noted in the BAA procedure using the GP method in a sample from India, and a huge error variability was observed for 5-year-old subjects of both genders. Typically, in the male and female samples between 3- and 6-year-old age groups, the skeletal retardation was in a range of 0.40–1.00 years. However, significant skeletal retardation occurred in the 4-year-old group (0.52 years for male subjects and 0.82 years for female subjects). Mansourvar et al. (21) concluded that the GP method is not reliable for various ethnic groups from all age groups except for Caucasian and Hispanic children. In the sample of Asian children, the skeletal age according to the GP method could be 2 years less than the CA in 4–6-year-old children. According to the present study, the GP method is not applicable in both genders because underestimation was reported in female subjects aged from 5 to 6 and male subjects aged from 4 to 6. In some specific samples, the Ds can be large than 2 years. After collecting 1,390 hand and wrist radiographs obtained in healthy children from Asian, African American, white, and Hispanic children, Zhang et al. (11) concluded that ethnic and racial differences in growth patterns exist at certain ages. When using the GP method to assess BA, it was significantly overestimated in Asian and Hispanic children. This result is the complete opposite. It is not clear whether the GP method can be used in the sample of Chinese during childhood.

Based on the results of the previous and current studies, the GP method should be used with caution in the different populations of preschool children.

### TW3 method

The TW3 method can evaluate and score the maturity of each hand and wrist bone (22). The reference children populations were from European and American families with average social economic status during the 1980s and 1990s, and the data were adjusted to the secular trend in 2001.

In recent years, several clinical studies (23–25) have demonstrated that the TW3 method is the most appropriate method showing the highest degree of accuracy in 3–4-year-old males. Despite having a tendency to underestimate the CA in subjects aged 5–6-year-old. The TW3 method was the most accurate among the methods tested.

Various studies have compared TW3 to GP method (24, 26). According to the investigation of the Turkish children, the TW3 method underestimated the age with a mean accuracy of 0.18 and 0.21 years for male and female subjects, respectively (24). Significant differences were detected between CA and BA in both sexes (*p* < 0.05). For males, the GP method was the most accurate, followed by the TW3 method, while for female individuals, the TW3 method was the most accurate, followed by the GP method. In the sample of normal Korean children, there was a lower absolute error of the TW3 method than that of the GP method for both genders (*p* < 0.05). The skeletal age using the TW3 and GP methods were overestimated for male individuals (59.6 and 54.6%) and female individuals (72.2 and 74.3%). However, in this study, the TW3 method showed the highest performance for male subjects and similar proficiency for female subjects compared to the GP and RUS–CHN methods.

As demonstrated above, the TW3 method is optimal for subjects between 5- and 6-year-old. TW3 can be regarded as the most reliable BAA method with the highest accuracy compared to the other two methods. However, the numerical accuracy is not remarkable, and our results are not consistent with previous findings (24, 26). When the TW3 method is used clinically, its impact on bone maturation should be considered.

### RUS–CHN method

In the past two decades, due to the trend of accelerated growth of Chinese children, Zhang et al. (9) have revised the standards based on the TW3 method and established the RUS–CHN method. The TW3 method is conducted on samples from European and American children, while RUS–CHN is a special BAA method designed for Chinese children. The RUS–CHN method selects new maturity indicators from some of the bone development levels in the TW3 method and divides the original into two levels. In addition, the RUS–CHN method divides the long-term fusion process of the radius and ulna into five grades for improved accuracy (27). Several studies have been carried out on this BAA method (12, 28–30).

According to a study for Chinese children, the RUS–CHN method can be used in all age groups of Han nationality. It showed an accuracy in BAA similar to the TW3 method. After analyzing 2,438 radiographs of 1,137 male and 1,301 female subjects, the differences between the CA and BA of each age group were 0.0–0.3 years in the RUS–CHN and TW3 bone maturity standards.

Recently, Xiong et al. (30) concluded that the RUS–CHN method was reliable for children of Han Nationality and applicable for children of Uygur nationality for various ages, especially in samples of 4.0–9.9-year-old age group. A good consistency was observed between the RUS–CHN and the TW3 methods in 3–6-year-old girls.

In the study by Zhang et al. (31), the radiographs of 45 children were assessed by 13 observers, who demonstrated the advanced reliability of RUS–CHN method in BAA. The study revealed that interobserver percentage agreement of ratings ranged from 70.5 to 92.1% (mean 84.3%) and 69.0–83.3% (mean 78.1%), respectively, and the 95% confidence limits for a single reading were ± 0.26 to ± 0.49 and ± 0.35 to ± 0.56, respectively.

In the present study, the RUS–CHN method shows maximal Ds among the three BAA methods. The prediction of BA is underestimated in each age group of both genders, especially in male individuals. Despite the RUS–CHN method having the advantages of high accuracy and repeatability, it showed an overall overestimation tendency and a false positive rate when applying for BAA in preschool children.

The present study has some limitations. First, this is a cross-sectional study using single-center data, and therefore the sample size was small. Second, it only covered a specific range of the population in the Zhejiang Province without including other areas in China. These results may be applicable only to the urban area of the Zhejiang Province or metropolitan areas with similar development statuses. Third, age groups under 3-year-old were excluded in both sexes. Finally, we could not ascertain full health in children, although no remarkable medical history for any genetic syndrome or trauma was noted in the hospital records. Hence, in-depth multicenter studies are essential to substantiate these findings.

## Conclusion

The present study aimed to compare the CA and BA of Chinese preschool-aged children using the GP, TW3, and RUS–CHN methods to determine the reliability. Based on the results, we could draw the following conclusions: (i) the GP, TW3, and RUS–CHN methods are reliable for BAA in clinical practice for selected age classes (e.g., TW3 in 6-year-old); (ii) TW3 is the most suitable method to predict the BA of males, with the highest accuracy among the three methods (TW3: 32.24%, GP: 12.02%, RUS–CHN: 12.02%); (iii) the GP, TW3, and RUS–CHN methods have a low rate of correct classification in females (GP: 25.76%, TW3: 24.15%, and RUS–CHN: 21.26%); (iv) the GP method is not accurate due to its remarkable trend to underestimate the BA, especially in males; (v) the TW3 and RUS–CHN methods are not accurate, given the overestimation of BAA for both genders; and (vi) predictions within 6 months (CA ± 90 days) can rarely provide the correct classification results.

In conclusion, this study demonstrated that the results of BAA using the TW3 method were more reliable than the GP and RUS–CHN methods. Until an effective method is developed, we proposed that TW3 be utilized. However, added diagnostic value is provided by combining multiple methods when assessing BA. Due to the diagnostic value of BAA, improving the accuracy of BA methods is crucial to guide timely pediatric treatments.

## Data availability statement

The original contributions presented in this study are included in the article/Supplementary material, further inquiries can be directed to the corresponding authors.

## Ethics statement

Written informed consent was obtained from the minor(s)’ legal guardian/next of kin for the publication of any potentially identifiable images or data included in this article.

## Author contributions

CG, ML, and ZD conceived and designed the study, had full access to the data in the study and took responsibility for the integrity of the data and the accuracy of data analysis. CG, QQ, ML, and ZD drafted the manuscript. CG, QQ, XX, and XH analyzed the data. CG, YL, and XX contributed to data acquisition. All authors critically revised the manuscript for intellectual content and approved the version for publication, agreed to be accountable for all aspects of the work related to the accuracy or integrity for appropriate investigation and resolution of any queries.
