# Supplementary material for: A comparative study of three bone age assessment methods on Chinese preschool-aged children
Source: Front Pediatr. 2022 Aug 16;10:976565. doi: 10.3389/fped.2022.976565 (PMC9424682; doi:10.3389/fped.2022.976565)
Supplement: Supplementary file 1 [file Data_Sheet_1.PDF]

| number | sex | CA | R1_TW3 | R2_TW3 | average_TW3 |
|---|---|---|---|---|---|
| 1 | F | 3.000 | 4.667 | 4.833 | 4.750 |
| 2 | F | 3.000 | 4.083 | 4.083 | 4.083 |
| 3 | F | 3.000 | 4.167 | 4.167 | 4.167 |
| 4 | F | 3.083 | 4.500 | 4.500 | 4.500 |
| 5 | F | 3.083 | 4.000 | 4.167 | 4.084 |
| 6 | F | 3.167 | 4.167 | 4.167 | 4.167 |
| 7 | F | 3.250 | 4.833 | 4.833 | 4.833 |
| 8 | F | 3.333 | 4.500 | 4.500 | 4.500 |
| 9 | F | 3.333 | 3.500 | 3.000 | 3.250 |
| 10 | F | 3.417 | 4.500 | 4.500 | 4.500 |
| 11 | F | 3.417 | 4.083 | 4.083 | 4.083 |
| 12 | F | 3.500 | 3.750 | 3.583 | 3.667 |
| 13 | F | 3.500 | 3.833 | 3.833 | 3.833 |
| 14 | F | 3.500 | 4.083 | 4.083 | 4.083 |
| 15 | F | 3.583 | 5.583 | 5.583 | 5.583 |
| 16 | F | 3.667 | 4.000 | 4.167 | 4.084 |
| 17 | F | 3.667 | 5.083 | 5.250 | 5.167 |
| 18 | F | 3.667 | 4.167 | 4.167 | 4.167 |
| 19 | F | 3.750 | 4.083 | 4.083 | 4.083 |
| 20 | F | 3.833 | 4.250 | 4.750 | 4.500 |
| 21 | F | 3.833 | 5.083 | 5.083 | 5.083 |
| 22 | F | 3.917 | 5.083 | 5.083 | 5.083 |
| 23 | F | 3.917 | 4.500 | 4.500 | 4.500 |
| 24 | F | 3.917 | 5.250 | 5.417 | 5.334 |
| 25 | F | 4.000 | 5.250 | 5.417 | 5.334 |
| 26 | F | 4.083 | 5.833 | 5.833 | 5.833 |
| 27 | F | 4.083 | 5.000 | 5.000 | 5.000 |
| 28 | F | 4.083 | 4.833 | 4.833 | 4.833 |
| 29 | F | 4.083 | 5.000 | 4.833 | 4.917 |
| 30 | F | 4.167 | 4.750 | 4.750 | 4.750 |
| 31 | F | 4.250 | 5.083 | 5.083 | 5.083 |
| 32 | F | 4.250 | 5.083 | 5.083 | 5.083 |
| 33 | F | 4.250 | 5.500 | 5.667 | 5.584 |
| 34 | F | 4.250 | 4.583 | 4.583 | 4.583 |
| 35 | F | 4.250 | 4.417 | 4.417 | 4.417 |
| 36 | F | 4.250 | 6.000 | 6.167 | 6.084 |
| 37 | F | 4.333 | 4.000 | 4.000 | 4.000 |
| 38 | F | 4.417 | 5.000 | 4.667 | 4.834 |
| 39 | F | 4.500 | 4.917 | 4.917 | 4.917 |
| 40 | F | 4.500 | 4.083 | 4.083 | 4.083 |
| 41 | F | 4.500 | 5.083 | 5.250 | 5.167 |
| 42 | F | 4.583 | 5.000 | 5.000 | 5.000 |
| 43 | F | 4.583 | 5.500 | 5.500 | 5.500 |
| 44 | F | 4.583 | 4.667 | 4.667 | 4.667 |
| 45 | F | 4.667 | 6.500 | 6.333 | 6.417 |
| 46 | F | 4.667 | 5.000 | 5.000 | 5.000 |
| 47 | F | 4.667 | 5.750 | 5.750 | 5.750 |
| 48 | F | 4.667 | 5.333 | 5.333 | 5.333 |
| 49 | F | 4.750 | 5.417 | 5.583 | 5.500 |
| 50 | F | 4.750 | 5.750 | 5.083 | 5.417 |
| 51 | F | 4.750 | 5.750 | 5.750 | 5.750 |
| 52 | F | 4.750 | 4.917 | 4.917 | 4.917 |
| 53 | F | 4.750 | 5.250 | 4.750 | 5.000 |
| 54 | F | 4.750 | 5.083 | 5.083 | 5.083 |
| 55 | F | 4.833 | 5.750 | 5.750 | 5.750 |
| 56 | F | 4.833 | 5.583 | 5.583 | 5.583 |
| 57 | F | 4.917 | 5.583 | 5.583 | 5.583 |

| | | | | | |
|---|---|---|---|---|---|
| 58 | F | 4.917 | 6.583 | 6.583 | 6.583 |
| 59 | F | 4.917 | 6.167 | 6.167 | 6.167 |
| 60 | F | 4.917 | 3.500 | 4.000 | 3.750 |
| 61 | F | 5.000 | 5.333 | 5.333 | 5.333 |
| 62 | F | 5.000 | 5.583 | 5.583 | 5.583 |
| 63 | F | 5.000 | 4.583 | 4.583 | 4.583 |
| 64 | F | 5.000 | 4.583 | 4.583 | 4.583 |
| 65 | F | 5.000 | 5.083 | 5.250 | 5.167 |
| 66 | F | 5.083 | 5.500 | 5.500 | 5.500 |
| 67 | F | 5.083 | 5.583 | 5.583 | 5.583 |
| 68 | F | 5.083 | 5.583 | 5.583 | 5.583 |
| 69 | F | 5.083 | 5.500 | 5.667 | 5.584 |
| 70 | F | 5.083 | 4.167 | 4.167 | 4.167 |
| 71 | F | 5.083 | 4.833 | 4.833 | 4.833 |
| 72 | F | 5.083 | 5.583 | 5.583 | 5.583 |
| 73 | F | 5.083 | 5.500 | 5.667 | 5.584 |
| 74 | F | 5.167 | 5.583 | 5.583 | 5.583 |
| 75 | F | 5.167 | 5.333 | 5.333 | 5.333 |
| 76 | F | 5.167 | 5.333 | 5.333 | 5.333 |
| 77 | F | 5.250 | 5.750 | 5.250 | 5.500 |
| 78 | F | 5.250 | 5.750 | 5.750 | 5.750 |
| 79 | F | 5.250 | 5.750 | 5.750 | 5.750 |
| 80 | F | 5.250 | 5.000 | 5.000 | 5.000 |
| 81 | F | 5.333 | 6.000 | 5.667 | 5.834 |
| 82 | F | 5.417 | 6.083 | 6.083 | 6.083 |
| 83 | F | 5.417 | 6.333 | 6.333 | 6.333 |
| 84 | F | 5.417 | 6.000 | 5.000 | 5.500 |
| 85 | F | 5.417 | 5.583 | 5.583 | 5.583 |
| 86 | F | 5.417 | 5.500 | 5.500 | 5.500 |
| 87 | F | 5.417 | 6.667 | 6.833 | 6.750 |
| 88 | F | 5.417 | 5.583 | 5.583 | 5.583 |
| 89 | F | 5.417 | 5.500 | 5.500 | 5.500 |
| 90 | F | 5.500 | 5.667 | 5.833 | 5.750 |
| 91 | F | 5.500 | 6.333 | 6.333 | 6.333 |
| 92 | F | 5.500 | 6.083 | 6.083 | 6.083 |
| 93 | F | 5.500 | 6.083 | 6.083 | 6.083 |
| 94 | F | 5.500 | 6.583 | 6.583 | 6.583 |
| 95 | F | 5.583 | 5.583 | 5.750 | 5.667 |
| 96 | F | 5.583 | 7.000 | 7.333 | 7.167 |
| 97 | F | 5.583 | 6.000 | 6.000 | 6.000 |
| 98 | F | 5.583 | 6.583 | 6.583 | 6.583 |
| 99 | F | 5.667 | 6.167 | 6.167 | 6.167 |
| 100 | F | 5.667 | 5.750 | 5.917 | 5.834 |
| 101 | F | 5.667 | 5.750 | 5.750 | 5.750 |
| 102 | F | 5.667 | 5.750 | 5.750 | 5.750 |
| 103 | F | 5.667 | 5.750 | 5.750 | 5.750 |
| 104 | F | 5.667 | 7.000 | 7.167 | 7.084 |
| 105 | F | 5.750 | 6.000 | 6.167 | 6.084 |
| 106 | F | 5.750 | 6.083 | 6.083 | 6.083 |
| 107 | F | 5.750 | 5.583 | 5.583 | 5.583 |
| 108 | F | 5.750 | 6.083 | 6.083 | 6.083 |
| 109 | F | 5.750 | 6.000 | 6.000 | 6.000 |
| 110 | F | 5.833 | 5.500 | 6.000 | 5.750 |
| 111 | F | 5.833 | 6.083 | 6.083 | 6.083 |
| 112 | F | 5.833 | 6.750 | 6.750 | 6.750 |
| 113 | F | 5.833 | 6.250 | 6.250 | 6.250 |
| 114 | F | 5.833 | 6.083 | 6.250 | 6.167 |
| 115 | F | 5.833 | 5.500 | 5.500 | 5.500 |

| | | | | | |
|---|---|---|---|---|---|
| 116 | F | 5.833 | 5.833 | 5.833 | 5.833 |
| 117 | F | 5.833 | 6.250 | 6.083 | 6.167 |
| 118 | F | 5.833 | 6.250 | 6.083 | 6.167 |
| 119 | F | 5.917 | 6.000 | 6.167 | 6.084 |
| 120 | F | 5.917 | 6.833 | 6.833 | 6.833 |
| 121 | F | 5.917 | 6.083 | 6.083 | 6.083 |
| 122 | F | 5.917 | 6.167 | 6.167 | 6.167 |
| 123 | F | 5.917 | 6.000 | 6.167 | 6.084 |
| 124 | F | 5.917 | 6.167 | 6.167 | 6.167 |
| 125 | F | 5.917 | 6.167 | 6.167 | 6.167 |
| 126 | F | 5.917 | 6.500 | 6.500 | 6.500 |
| 127 | F | 5.917 | 5.083 | 5.250 | 5.167 |
| 128 | F | 6.000 | 6.000 | 6.167 | 6.084 |
| 129 | F | 6.000 | 6.167 | 6.000 | 6.084 |
| 130 | F | 6.000 | 5.833 | 5.833 | 5.833 |
| 131 | F | 6.000 | 6.250 | 6.250 | 6.250 |
| 132 | F | 6.000 | 5.583 | 5.583 | 5.583 |
| 133 | F | 6.000 | 7.000 | 7.167 | 7.084 |
| 134 | F | 6.000 | 5.000 | 4.667 | 4.834 |
| 135 | F | 6.000 | 6.250 | 6.250 | 6.250 |
| 136 | F | 6.000 | 9.167 | 9.167 | 9.167 |
| 137 | F | 6.083 | 7.083 | 7.083 | 7.083 |
| 138 | F | 6.083 | 6.500 | 7.000 | 6.750 |
| 139 | F | 6.083 | 6.667 | 7.000 | 6.834 |
| 140 | F | 6.083 | 5.833 | 5.833 | 5.833 |
| 141 | F | 6.083 | 6.000 | 6.000 | 6.000 |
| 142 | F | 6.083 | 8.667 | 8.667 | 8.667 |
| 143 | F | 6.083 | 7.500 | 7.667 | 7.584 |
| 144 | F | 6.167 | 6.417 | 7.083 | 6.750 |
| 145 | F | 6.167 | 5.750 | 5.750 | 5.750 |
| 146 | F | 6.167 | 5.583 | 5.583 | 5.583 |
| 147 | F | 6.167 | 6.083 | 6.083 | 6.083 |
| 148 | F | 6.167 | 7.750 | 7.917 | 7.834 |
| 149 | F | 6.167 | 5.250 | 5.083 | 5.167 |
| 150 | F | 6.167 | 6.000 | 6.000 | 6.000 |
| 151 | F | 6.167 | 5.500 | 5.500 | 5.500 |
| 152 | F | 6.167 | 6.500 | 6.500 | 6.500 |
| 153 | F | 6.250 | 6.500 | 7.167 | 6.834 |
| 154 | F | 6.250 | 5.000 | 5.000 | 5.000 |
| 155 | F | 6.250 | 7.833 | 7.833 | 7.833 |
| 156 | F | 6.250 | 5.000 | 5.000 | 5.000 |
| 157 | F | 6.250 | 5.500 | 6.000 | 5.750 |
| 158 | F | 6.250 | 5.750 | 5.750 | 5.750 |
| 159 | F | 6.250 | 5.083 | 5.083 | 5.083 |
| 160 | F | 6.250 | 7.167 | 7.167 | 7.167 |
| 161 | F | 6.250 | 8.000 | 8.333 | 8.167 |
| 162 | F | 6.333 | 5.583 | 5.583 | 5.583 |
| 163 | F | 6.333 | 5.333 | 5.333 | 5.333 |
| 164 | F | 6.333 | 5.750 | 5.750 | 5.750 |
| 165 | F | 6.417 | 7.000 | 7.167 | 7.084 |
| 166 | F | 6.417 | 7.250 | 6.917 | 7.084 |
| 167 | F | 6.417 | 7.083 | 7.083 | 7.083 |
| 168 | F | 6.417 | 7.000 | 7.000 | 7.000 |
| 169 | F | 6.417 | 6.833 | 6.833 | 6.833 |
| 170 | F | 6.417 | 7.250 | 6.750 | 7.000 |
| 171 | F | 6.417 | 5.583 | 5.583 | 5.583 |
| 172 | F | 6.417 | 6.167 | 6.167 | 6.167 |
| 173 | F | 6.417 | 6.000 | 6.167 | 6.084 |

| | | | | | |
|---|---|---|---|---|---|
| 174 | F | 6.417 | 6.500 | 6.667 | 6.584 |
| 175 | F | 6.417 | 5.000 | 5.167 | 5.084 |
| 176 | F | 6.500 | 7.000 | 7.000 | 7.000 |
| 177 | F | 6.500 | 7.083 | 7.083 | 7.083 |
| 178 | F | 6.500 | 7.583 | 7.583 | 7.583 |
| 179 | F | 6.583 | 7.250 | 6.750 | 7.000 |
| 180 | F | 6.583 | 5.750 | 5.750 | 5.750 |
| 181 | F | 6.583 | 5.583 | 5.583 | 5.583 |
| 182 | F | 6.583 | 7.500 | 7.667 | 7.584 |
| 183 | F | 6.583 | 5.667 | 5.667 | 5.667 |
| 184 | F | 6.667 | 8.250 | 8.250 | 8.250 |
| 185 | F | 6.667 | 7.500 | 7.000 | 7.250 |
| 186 | F | 6.667 | 7.167 | 7.167 | 7.167 |
| 187 | F | 6.667 | 7.500 | 7.500 | 7.500 |
| 188 | F | 6.667 | 6.833 | 6.833 | 6.833 |
| 189 | F | 6.667 | 6.250 | 6.083 | 6.167 |
| 190 | F | 6.750 | 7.250 | 7.250 | 7.250 |
| 191 | F | 6.750 | 5.583 | 5.583 | 5.583 |
| 192 | F | 6.750 | 7.667 | 7.667 | 7.667 |
| 193 | F | 6.750 | 5.000 | 4.667 | 4.834 |
| 194 | F | 6.750 | 7.667 | 7.667 | 7.667 |
| 195 | F | 6.833 | 7.167 | 7.167 | 7.167 |
| 196 | F | 6.833 | 7.250 | 7.250 | 7.250 |
| 197 | F | 6.833 | 7.500 | 6.500 | 7.000 |
| 198 | F | 6.833 | 7.000 | 7.500 | 7.250 |
| 199 | F | 6.833 | 5.500 | 5.667 | 5.584 |
| 200 | F | 6.833 | 6.333 | 6.333 | 6.333 |
| 201 | F | 6.917 | 7.000 | 7.000 | 7.000 |
| 202 | F | 6.917 | 5.833 | 5.833 | 5.833 |
| 203 | F | 6.917 | 5.500 | 5.667 | 5.584 |
| 204 | F | 6.917 | 8.583 | 8.583 | 8.583 |
| 205 | F | 6.917 | 7.083 | 7.083 | 7.083 |
| 206 | F | 6.917 | 7.000 | 7.333 | 7.167 |
| 207 | F | 6.917 | 9.500 | 8.500 | 9.000 |
| 1 | M | 3.000 | 3.167 | 3.333 | 3.250 |
| 2 | M | 3.083 | 3.250 | 3.250 | 3.250 |
| 3 | M | 3.083 | 4.500 | 4.500 | 4.500 |
| 4 | M | 3.250 | 3.250 | 3.250 | 3.250 |
| 5 | M | 3.250 | 4.000 | 4.167 | 4.084 |
| 6 | M | 3.250 | 3.000 | 3.167 | 3.084 |
| 7 | M | 3.333 | 5.000 | 4.833 | 4.917 |
| 8 | M | 3.333 | 4.000 | 3.833 | 3.917 |
| 9 | M | 3.417 | 3.000 | 3.167 | 3.084 |
| 10 | M | 3.417 | 4.000 | 4.000 | 4.000 |
| 11 | M | 3.500 | 5.250 | 5.250 | 5.250 |
| 12 | M | 3.500 | 4.333 | 4.333 | 4.333 |
| 13 | M | 3.583 | 4.000 | 4.167 | 4.084 |
| 14 | M | 3.667 | 4.917 | 4.917 | 4.917 |
| 15 | M | 3.667 | 5.000 | 5.167 | 5.084 |
| 16 | M | 3.667 | 4.667 | 4.667 | 4.667 |
| 17 | M | 3.667 | 4.500 | 4.500 | 4.500 |
| 18 | M | 3.750 | 5.167 | 5.167 | 5.167 |
| 19 | M | 3.750 | 4.000 | 4.667 | 4.334 |
| 20 | M | 3.833 | 3.000 | 3.333 | 3.167 |
| 21 | M | 3.917 | 4.333 | 4.333 | 4.333 |
| 22 | M | 3.917 | 5.333 | 5.333 | 5.333 |
| 23 | M | 3.917 | 4.500 | 5.000 | 4.750 |
| 24 | M | 3.917 | 4.583 | 4.583 | 4.583 |

| | | | | | |
|---|---|---|---|---|---|
| 25 | M | 4.000 | 4.750 | 4.750 | 4.750 |
| 26 | M | 4.000 | 6.667 | 6.667 | 6.667 |
| 27 | M | 4.000 | 4.000 | 4.000 | 4.000 |
| 28 | M | 4.000 | 4.000 | 4.167 | 4.084 |
| 29 | M | 4.000 | 4.250 | 4.250 | 4.250 |
| 30 | M | 4.000 | 5.167 | 5.167 | 5.167 |
| 31 | M | 4.083 | 4.333 | 4.667 | 4.500 |
| 32 | M | 4.083 | 4.167 | 4.167 | 4.167 |
| 33 | M | 4.083 | 4.167 | 4.167 | 4.167 |
| 34 | M | 4.167 | 4.083 | 4.083 | 4.083 |
| 35 | M | 4.250 | 5.000 | 5.000 | 5.000 |
| 36 | M | 4.250 | 6.500 | 6.500 | 6.500 |
| 37 | M | 4.250 | 5.000 | 4.833 | 4.917 |
| 38 | M | 4.250 | 4.750 | 4.750 | 4.750 |
| 39 | M | 4.250 | 4.750 | 4.750 | 4.750 |
| 40 | M | 4.250 | 4.500 | 4.500 | 4.500 |
| 41 | M | 4.250 | 4.833 | 4.667 | 4.750 |
| 42 | M | 4.250 | 4.917 | 4.917 | 4.917 |
| 43 | M | 4.333 | 6.667 | 6.683 | 6.675 |
| 44 | M | 4.417 | 4.000 | 4.000 | 4.000 |
| 45 | M | 4.417 | 4.167 | 4.167 | 4.167 |
| 46 | M | 4.500 | 4.833 | 4.833 | 4.833 |
| 47 | M | 4.500 | 4.583 | 4.583 | 4.583 |
| 48 | M | 4.500 | 5.000 | 5.000 | 5.000 |
| 49 | M | 4.500 | 5.333 | 5.333 | 5.333 |
| 50 | M | 4.500 | 4.667 | 4.667 | 4.667 |
| 51 | M | 4.583 | 4.583 | 4.583 | 4.583 |
| 52 | M | 4.583 | 4.667 | 5.000 | 4.834 |
| 53 | M | 4.583 | 4.500 | 4.500 | 4.500 |
| 54 | M | 4.583 | 6.167 | 6.000 | 6.084 |
| 55 | M | 4.667 | 4.333 | 4.333 | 4.333 |
| 56 | M | 4.667 | 6.500 | 6.500 | 6.500 |
| 57 | M | 4.667 | 5.000 | 4.833 | 4.917 |
| 58 | M | 4.667 | 4.917 | 4.917 | 4.917 |
| 59 | M | 4.667 | 6.000 | 5.833 | 5.917 |
| 60 | M | 4.667 | 5.917 | 5.917 | 5.917 |
| 61 | M | 4.750 | 5.833 | 5.833 | 5.833 |
| 62 | M | 4.833 | 5.000 | 5.000 | 5.000 |
| 63 | M | 4.833 | 5.000 | 5.000 | 5.000 |
| 64 | M | 4.833 | 5.250 | 5.250 | 5.250 |
| 65 | M | 4.833 | 6.083 | 6.083 | 6.083 |
| 66 | M | 4.917 | 4.583 | 4.583 | 4.583 |
| 67 | M | 4.917 | 6.250 | 6.250 | 6.250 |
| 68 | M | 4.917 | 5.250 | 5.250 | 5.250 |
| 69 | M | 4.917 | 4.833 | 4.833 | 4.833 |
| 70 | M | 4.917 | 5.000 | 5.000 | 5.000 |
| 71 | M | 5.000 | 5.000 | 5.000 | 5.000 |
| 72 | M | 5.000 | 5.500 | 5.500 | 5.500 |
| 73 | M | 5.083 | 6.000 | 6.000 | 6.000 |
| 74 | M | 5.083 | 7.583 | 7.583 | 7.583 |
| 75 | M | 5.083 | 5.333 | 5.333 | 5.333 |
| 76 | M | 5.083 | 5.083 | 5.083 | 5.083 |
| 77 | M | 5.167 | 6.250 | 6.250 | 6.250 |
| 78 | M | 5.167 | 5.000 | 5.000 | 5.000 |
| 79 | M | 5.167 | 5.750 | 5.750 | 5.750 |
| 80 | M | 5.250 | 5.333 | 5.333 | 5.333 |
| 81 | M | 5.250 | 5.500 | 5.500 | 5.500 |
| 82 | M | 5.250 | 5.500 | 5.500 | 5.500 |

| | | | | | |
|---|---|---|---|---|---|
| 83 | M | 5.333 | 5.333 | 5.333 | 5.333 |
| 84 | M | 5.333 | 5.667 | 5.833 | 5.750 |
| 85 | M | 5.333 | 5.500 | 5.500 | 5.500 |
| 86 | M | 5.417 | 5.583 | 5.583 | 5.583 |
| 87 | M | 5.417 | 6.083 | 6.083 | 6.083 |
| 88 | M | 5.417 | 6.583 | 6.750 | 6.667 |
| 89 | M | 5.417 | 6.250 | 6.250 | 6.250 |
| 90 | M | 5.417 | 5.833 | 5.833 | 5.833 |
| 91 | M | 5.500 | 5.667 | 5.833 | 5.750 |
| 92 | M | 5.500 | 6.333 | 6.333 | 6.333 |
| 93 | M | 5.583 | 5.250 | 5.250 | 5.250 |
| 94 | M | 5.667 | 5.833 | 5.833 | 5.833 |
| 95 | M | 5.667 | 6.583 | 6.583 | 6.583 |
| 96 | M | 5.750 | 6.250 | 6.250 | 6.250 |
| 97 | M | 5.750 | 5.750 | 5.750 | 5.750 |
| 98 | M | 5.750 | 5.833 | 5.833 | 5.833 |
| 99 | M | 5.833 | 7.167 | 7.167 | 7.167 |
| 100 | M | 5.833 | 6.250 | 6.250 | 6.250 |
| 101 | M | 5.833 | 6.000 | 6.167 | 6.084 |
| 102 | M | 5.833 | 6.000 | 6.000 | 6.000 |
| 103 | M | 5.833 | 6.000 | 6.000 | 6.000 |
| 104 | M | 5.833 | 6.167 | 6.167 | 6.167 |
| 105 | M | 5.833 | 6.000 | 6.000 | 6.000 |
| 106 | M | 5.833 | 6.167 | 6.333 | 6.250 |
| 107 | M | 5.917 | 6.000 | 6.000 | 6.000 |
| 108 | M | 5.917 | 6.333 | 6.333 | 6.333 |
| 109 | M | 5.917 | 6.167 | 6.167 | 6.167 |
| 110 | M | 6.000 | 6.250 | 6.250 | 6.250 |
| 111 | M | 6.000 | 7.000 | 7.000 | 7.000 |
| 112 | M | 6.000 | 6.333 | 6.333 | 6.333 |
| 113 | M | 6.000 | 5.000 | 5.000 | 5.000 |
| 114 | M | 6.000 | 6.667 | 6.667 | 6.667 |
| 115 | M | 6.000 | 6.667 | 6.667 | 6.667 |
| 116 | M | 6.083 | 6.583 | 6.417 | 6.500 |
| 117 | M | 6.083 | 7.833 | 7.833 | 7.833 |
| 118 | M | 6.083 | 6.667 | 6.667 | 6.667 |
| 119 | M | 6.083 | 5.833 | 5.833 | 5.833 |
| 120 | M | 6.083 | 6.500 | 6.500 | 6.500 |
| 121 | M | 6.083 | 6.333 | 6.333 | 6.333 |
| 122 | M | 6.083 | 6.333 | 6.333 | 6.333 |
| 123 | M | 6.167 | 6.667 | 6.667 | 6.667 |
| 124 | M | 6.167 | 6.750 | 6.750 | 6.750 |
| 125 | M | 6.167 | 6.667 | 6.667 | 6.667 |
| 126 | M | 6.167 | 6.750 | 6.750 | 6.750 |
| 127 | M | 6.167 | 6.000 | 6.000 | 6.000 |
| 128 | M | 6.167 | 7.000 | 7.167 | 7.084 |
| 129 | M | 6.250 | 6.750 | 6.750 | 6.750 |
| 130 | M | 6.250 | 7.917 | 7.750 | 7.834 |
| 131 | M | 6.250 | 6.750 | 6.750 | 6.750 |
| 132 | M | 6.250 | 7.083 | 7.083 | 7.083 |
| 133 | M | 6.250 | 5.083 | 5.083 | 5.083 |
| 134 | M | 6.250 | 7.583 | 7.583 | 7.583 |
| 135 | M | 6.333 | 6.667 | 6.667 | 6.667 |
| 136 | M | 6.333 | 6.167 | 6.167 | 6.167 |
| 137 | M | 6.333 | 5.667 | 5.833 | 5.750 |
| 138 | M | 6.333 | 5.667 | 5.667 | 5.667 |
| 139 | M | 6.333 | 6.583 | 6.750 | 6.667 |
| 140 | M | 6.417 | 7.167 | 7.167 | 7.167 |

| | | | | | |
|---|---|---|---|---|---|
| 141 | M | 6.417 | 6.500 | 7.000 | 6.750 |
| 142 | M | 6.417 | 6.833 | 6.833 | 6.833 |
| 143 | M | 6.417 | 7.083 | 7.083 | 7.083 |
| 144 | M | 6.417 | 6.667 | 6.667 | 6.667 |
| 145 | M | 6.500 | 7.000 | 7.000 | 7.000 |
| 146 | M | 6.500 | 7.167 | 7.167 | 7.167 |
| 147 | M | 6.500 | 8.167 | 8.500 | 8.334 |
| 148 | M | 6.500 | 6.083 | 5.917 | 6.000 |
| 149 | M | 6.500 | 5.250 | 5.250 | 5.250 |
| 150 | M | 6.583 | 6.417 | 6.250 | 6.334 |
| 151 | M | 6.583 | 7.000 | 7.167 | 7.084 |
| 152 | M | 6.583 | 5.833 | 5.833 | 5.833 |
| 153 | M | 6.667 | 5.667 | 5.667 | 5.667 |
| 154 | M | 6.667 | 7.583 | 7.583 | 7.583 |
| 155 | M | 6.667 | 6.750 | 6.750 | 6.750 |
| 156 | M | 6.667 | 6.667 | 6.667 | 6.667 |
| 157 | M | 6.667 | 7.833 | 7.833 | 7.833 |
| 158 | M | 6.667 | 7.167 | 7.167 | 7.167 |
| 159 | M | 6.667 | 6.833 | 6.667 | 6.750 |
| 160 | M | 6.667 | 6.250 | 6.250 | 6.250 |
| 161 | M | 6.750 | 6.333 | 6.333 | 6.333 |
| 162 | M | 6.750 | 7.333 | 7.333 | 7.333 |
| 163 | M | 6.750 | 7.750 | 7.750 | 7.750 |
| 164 | M | 6.750 | 6.167 | 6.333 | 6.250 |
| 165 | M | 6.750 | 6.667 | 6.667 | 6.667 |
| 166 | M | 6.750 | 6.000 | 5.500 | 5.750 |
| 167 | M | 6.750 | 7.083 | 7.083 | 7.083 |
| 168 | M | 6.750 | 5.833 | 5.833 | 5.833 |
| 169 | M | 6.833 | 7.000 | 7.167 | 7.084 |
| 170 | M | 6.833 | 6.667 | 6.667 | 6.667 |
| 171 | M | 6.833 | 7.167 | 7.167 | 7.167 |
| 172 | M | 6.833 | 7.167 | 7.167 | 7.167 |
| 173 | M | 6.833 | 7.083 | 7.083 | 7.083 |
| 174 | M | 6.833 | 7.167 | 7.167 | 7.167 |
| 175 | M | 6.833 | 7.000 | 7.333 | 7.167 |
| 176 | M | 6.833 | 7.167 | 7.167 | 7.167 |
| 177 | M | 6.917 | 6.250 | 6.250 | 6.250 |
| 178 | M | 6.917 | 6.750 | 6.750 | 6.750 |
| 179 | M | 6.917 | 5.500 | 5.500 | 5.500 |
| 180 | M | 6.917 | 6.250 | 6.250 | 6.250 |
| 181 | M | 6.917 | 5.167 | 5.500 | 5.334 |
| 182 | M | 6.917 | 7.417 | 7.583 | 7.500 |
| 183 | M | 6.917 | 6.417 | 6.583 | 6.500 |

| R1_RUS-CHN | R2_RUS-CHN | average_RUS-CHN | R1_GP | R2_GP |
|---|---|---|---|---|
| 5.000 | 4.833 | 4.917 | 3.500 | 3.500 |
| 5.083 | 5.083 | 5.083 | 3.500 | 3.500 |
| 4.917 | 4.917 | 4.917 | 3.500 | 4.167 |
| 4.667 | 4.667 | 4.667 | 3.000 | 3.500 |
| 4.750 | 4.917 | 4.834 | 2.000 | 2.500 |
| 3.917 | 3.917 | 3.917 | 3.500 | 3.500 |
| 5.000 | 5.000 | 5.000 | 4.167 | 4.167 |
| 4.917 | 4.917 | 4.917 | 2.500 | 2.500 |
| 5.000 | 4.833 | 4.917 | 3.500 | 3.500 |
| 5.083 | 5.083 | 5.083 | 4.167 | 4.167 |
| 4.917 | 4.917 | 4.917 | 3.000 | 3.000 |
| 5.333 | 5.333 | 5.333 | 3.500 | 3.500 |
| 5.167 | 5.333 | 5.250 | 3.000 | 3.000 |
| 4.750 | 4.750 | 4.750 | 4.167 | 4.167 |
| 3.667 | 3.667 | 3.667 | 4.167 | 4.167 |
| 3.750 | 7.917 | 5.834 | 4.167 | 4.167 |
| 3.250 | 3.750 | 3.500 | 3.500 | 4.167 |
| 5.250 | 5.083 | 5.167 | 3.500 | 3.500 |
| 5.167 | 5.167 | 5.167 | 5.000 | 5.000 |
| 5.500 | 5.500 | 5.500 | 3.500 | 3.500 |
| 4.833 | 4.833 | 4.833 | 4.167 | 4.167 |
| 4.167 | 4.167 | 4.167 | 3.500 | 4.167 |
| 4.083 | 4.083 | 4.083 | 3.500 | 5.000 |
| 4.583 | 4.583 | 4.583 | 5.000 | 5.000 |
| 5.333 | 5.500 | 5.417 | 4.167 | 4.167 |
| 6.333 | 6.333 | 6.333 | 5.750 | 5.750 |
| 5.583 | 5.583 | 5.583 | 5.000 | 5.000 |
| 5.333 | 5.333 | 5.333 | 4.167 | 5.000 |
| 5.250 | 5.250 | 5.250 | 3.500 | 5.750 |
| 4.917 | 4.917 | 4.917 | 3.000 | 3.000 |
| 5.250 | 5.250 | 5.250 | 5.000 | 4.167 |
| 5.167 | 5.333 | 5.250 | 5.000 | 5.000 |
| 6.000 | 6.167 | 6.084 | 5.750 | 5.000 |
| 5.000 | 5.000 | 5.000 | 4.167 | 4.167 |
| 5.167 | 5.167 | 5.167 | 5.750 | 3.000 |
| 6.500 | 7.000 | 6.750 | 5.000 | 5.750 |
| 4.083 | 4.083 | 4.083 | 3.000 | 3.500 |
| 5.750 | 5.250 | 5.500 | 4.167 | 5.000 |
| 5.000 | 5.000 | 5.000 | 4.167 | 5.750 |
| 5.500 | 5.500 | 5.500 | 3.500 | 4.167 |
| 5.333 | 5.500 | 5.417 | 5.000 | 4.167 |
| 5.000 | 5.167 | 5.084 | 3.000 | 6.833 |
| 4.833 | 5.000 | 4.917 | 3.500 | 3.500 |
| 5.250 | 5.250 | 5.250 | 4.167 | 4.167 |
| 6.750 | 6.750 | 6.750 | 5.750 | 5.750 |
| 5.583 | 5.583 | 5.583 | 5.000 | 4.167 |
| 6.250 | 6.250 | 6.250 | 5.000 | 5.000 |
| 5.917 | 5.917 | 5.917 | 3.500 | 5.750 |
| 5.417 | 5.583 | 5.500 | 5.000 | 4.167 |
| 6.000 | 6.167 | 6.084 | 5.000 | 5.750 |
| 6.167 | 6.167 | 6.167 | 5.750 | 5.750 |
| 5.000 | 5.000 | 5.000 | 4.167 | 5.000 |
| 5.417 | 5.417 | 5.417 | 4.167 | 5.000 |
| 5.500 | 5.500 | 5.500 | 4.167 | 4.167 |
| 6.417 | 6.417 | 6.417 | 4.167 | 6.833 |
| 6.833 | 6.833 | 6.833 | 5.750 | 5.000 |
| 5.667 | 5.667 | 5.667 | 3.500 | 4.167 |

| | | | | |
|---|---|---|---|---|
| 7.083 | 7.083 | 7.083 | 4.167 | 5.000 |
| 6.417 | 6.417 | 6.417 | 3.500 | 3.500 |
| 5.750 | 5.917 | 5.834 | 3.000 | 3.000 |
| 5.500 | 5.333 | 5.417 | 5.000 | 5.000 |
| 5.667 | 5.667 | 5.667 | 5.750 | 5.750 |
| 4.667 | 4.667 | 4.667 | 3.500 | 3.500 |
| 4.750 | 4.750 | 4.750 | 3.000 | 3.500 |
| 5.583 | 5.583 | 5.583 | 3.500 | 3.500 |
| 5.500 | 5.500 | 5.500 | 5.000 | 5.000 |
| 5.667 | 5.667 | 5.667 | 4.167 | 4.167 |
| 5.667 | 5.667 | 5.667 | 4.167 | 5.000 |
| 5.500 | 5.833 | 5.667 | 4.167 | 5.000 |
| 4.500 | 4.500 | 4.500 | 3.500 | 3.500 |
| 5.667 | 5.667 | 5.667 | 3.500 | 4.167 |
| 5.667 | 5.667 | 5.667 | 5.000 | 5.000 |
| 5.667 | 5.667 | 5.667 | 3.500 | 4.167 |
| 5.667 | 5.667 | 5.667 | 4.167 | 4.167 |
| 5.667 | 5.667 | 5.667 | 4.167 | 4.167 |
| 5.417 | 5.417 | 5.417 | 4.167 | 4.167 |
| 5.500 | 5.500 | 5.500 | 4.167 | 4.167 |
| 5.750 | 6.000 | 5.875 | 4.167 | 4.167 |
| 5.833 | 5.833 | 5.833 | 5.000 | 5.000 |
| 5.167 | 5.167 | 5.167 | 3.500 | 4.167 |
| 6.000 | 6.000 | 6.000 | 5.750 | 4.160 |
| 6.250 | 6.250 | 6.250 | 5.000 | 5.000 |
| 6.667 | 6.667 | 6.667 | 5.000 | 5.750 |
| 5.500 | 5.667 | 5.584 | 4.167 | 5.000 |
| 5.583 | 5.750 | 5.667 | 4.167 | 4.167 |
| 5.500 | 5.500 | 5.500 | 5.000 | 5.000 |
| 6.750 | 6.750 | 6.750 | 6.833 | 5.750 |
| 5.667 | 5.667 | 5.667 | 4.167 | 5.000 |
| 5.583 | 5.583 | 5.583 | 4.167 | 4.167 |
| 5.833 | 5.833 | 5.833 | 5.000 | 5.000 |
| 6.417 | 6.417 | 6.417 | 5.750 | 5.750 |
| 6.083 | 6.333 | 6.208 | 5.000 | 5.000 |
| 6.667 | 6.667 | 6.667 | 6.833 | 5.750 |
| 7.917 | 7.917 | 7.917 | 5.750 | 5.750 |
| 5.500 | 5.833 | 5.667 | 4.167 | 4.167 |
| 7.500 | 7.667 | 7.584 | 6.833 | 6.833 |
| 6.167 | 6.167 | 6.167 | 5.000 | 5.000 |
| 7.083 | 7.083 | 7.083 | 6.833 | 5.750 |
| 7.500 | 7.500 | 7.500 | 5.000 | 5.750 |
| 6.250 | 5.750 | 6.000 | 5.000 | 5.000 |
| 6.000 | 6.000 | 6.000 | 5.750 | 5.750 |
| 5.833 | 5.833 | 5.833 | 5.750 | 5.750 |
| 5.833 | 5.833 | 5.833 | 5.750 | 5.750 |
| 7.750 | 7.583 | 7.667 | 6.833 | 6.833 |
| 6.083 | 6.417 | 6.250 | 5.750 | 5.750 |
| 6.250 | 6.083 | 6.167 | 4.167 | 4.167 |
| 5.667 | 5.667 | 5.667 | 5.000 | 5.000 |
| 6.167 | 6.167 | 6.167 | 5.000 | 5.750 |
| 6.000 | 6.000 | 6.000 | 5.000 | 5.000 |
| 5.833 | 5.833 | 5.833 | 5.000 | 5.000 |
| 6.167 | 6.167 | 6.167 | 5.000 | 5.000 |
| 5.833 | 5.833 | 5.833 | 4.167 | 5.000 |
| 6.583 | 6.583 | 6.583 | 5.750 | 5.750 |
| 6.333 | 6.500 | 6.417 | 5.750 | 5.750 |
| 5.500 | 5.667 | 5.584 | 5.000 | 5.000 |

| | | | | |
|---|---|---|---|---|
| 6.083 | 5.917 | 6.000 | 4.167 | 4.167 |
| 6.750 | 6.417 | 6.584 | 5.000 | 5.750 |
| 6.833 | 6.667 | 6.750 | 5.750 | 6.833 |
| 6.167 | 6.167 | 6.167 | 5.750 | 5.750 |
| 6.917 | 6.917 | 6.917 | 6.833 | 5.750 |
| 6.167 | 6.167 | 6.167 | 5.750 | 5.750 |
| 6.750 | 6.750 | 6.750 | 5.750 | 5.750 |
| 6.500 | 6.500 | 6.500 | 5.000 | 5.000 |
| 6.167 | 6.167 | 6.167 | 6.833 | 4.167 |
| 6.500 | 6.500 | 6.500 | 5.750 | 5.750 |
| 6.417 | 6.583 | 6.500 | 5.000 | 5.750 |
| 6.250 | 6.417 | 6.334 | 3.500 | 4.167 |
| 6.167 | 6.167 | 6.167 | 5.750 | 5.750 |
| 6.167 | 6.167 | 6.167 | 5.000 | 5.000 |
| 6.000 | 6.000 | 6.000 | 5.750 | 5.000 |
| 6.500 | 6.333 | 6.417 | 5.750 | 5.750 |
| 5.667 | 5.667 | 5.667 | 5.000 | 5.000 |
| 7.083 | 7.083 | 7.083 | 5.750 | 5.750 |
| 5.000 | 5.000 | 5.000 | 3.500 | 3.000 |
| 6.667 | 6.667 | 6.667 | 5.750 | 5.750 |
| 9.500 | 9.500 | 9.500 | 8.833 | 8.833 |
| 7.583 | 7.583 | 7.583 | 5.750 | 6.833 |
| 7.000 | 7.000 | 7.000 | 5.000 | 6.833 |
| 7.250 | 7.750 | 7.500 | 5.750 | 6.833 |
| 6.000 | 6.000 | 6.000 | 5.750 | 5.750 |
| 6.000 | 6.000 | 6.000 | 5.750 | 6.833 |
| 8.667 | 8.667 | 8.667 | 5.750 | 5.750 |
| 7.833 | 7.833 | 7.833 | 5.750 | 6.833 |
| 6.583 | 6.917 | 6.750 | 5.750 | 5.750 |
| 6.083 | 5.917 | 6.000 | 5.750 | 5.750 |
| 5.667 | 5.667 | 5.667 | 5.000 | 4.167 |
| 6.167 | 6.167 | 6.167 | 5.000 | 4.167 |
| 8.417 | 8.417 | 8.417 | 7.833 | 6.833 |
| 5.417 | 5.083 | 5.250 | 4.167 | 4.167 |
| 6.417 | 6.417 | 6.417 | 5.750 | 5.750 |
| 5.667 | 5.667 | 5.667 | 4.167 | 4.167 |
| 7.167 | 7.167 | 7.167 | 6.833 | 6.833 |
| 7.333 | 7.333 | 7.333 | 6.833 | 6.833 |
| 5.500 | 4.833 | 5.167 | 3.500 | 3.500 |
| 8.250 | 7.750 | 8.000 | 6.833 | 6.833 |
| 5.083 | 5.250 | 5.167 | 4.167 | 4.167 |
| 5.917 | 5.750 | 5.834 | 5.000 | 5.750 |
| 5.750 | 5.917 | 5.834 | 5.750 | 5.750 |
| 5.083 | 5.250 | 5.167 | 3.500 | 3.500 |
| 7.750 | 7.750 | 7.750 | 5.750 | 6.833 |
| 8.417 | 8.417 | 8.417 | 6.833 | 6.833 |
| 5.667 | 5.667 | 5.667 | 5.750 | 5.750 |
| 5.417 | 5.417 | 5.417 | 5.000 | 5.000 |
| 6.000 | 6.000 | 6.000 | 5.750 | 5.750 |
| 7.000 | 7.000 | 7.000 | 5.750 | 6.833 |
| 7.250 | 7.417 | 7.334 | 6.833 | 6.833 |
| 7.000 | 7.167 | 7.084 | 6.833 | 6.833 |
| 7.750 | 7.750 | 7.750 | 5.750 | 5.750 |
| 6.500 | 6.500 | 6.500 | 5.833 | 5.833 |
| 7.500 | 7.000 | 7.250 | 6.833 | 6.833 |
| 5.667 | 5.667 | 5.667 | 5.750 | 5.750 |
| 6.250 | 6.250 | 6.250 | 5.000 | 5.000 |
| 6.167 | 6.167 | 6.167 | 5.000 | 5.000 |

| | | | | |
|---|---|---|---|---|
| 6.417 | 6.583 | 6.500 | 5.000 | 5.750 |
| 5.583 | 5.583 | 5.583 | 5.000 | 5.000 |
| 7.083 | 7.083 | 7.083 | 5.750 | 7.833 |
| 7.333 | 7.333 | 7.333 | 5.750 | 6.833 |
| 7.833 | 7.833 | 7.833 | 6.833 | 6.833 |
| 7.250 | 7.250 | 7.250 | 6.833 | 7.833 |
| 5.750 | 5.917 | 5.834 | 5.000 | 5.000 |
| 5.833 | 5.500 | 5.667 | 3.500 | 4.167 |
| 7.833 | 8.000 | 7.917 | 6.833 | 7.833 |
| 5.667 | 5.667 | 5.667 | 4.167 | 5.000 |
| 8.750 | 8.750 | 8.750 | 7.833 | 7.833 |
| 8.000 | 8.000 | 8.000 | 6.833 | 7.833 |
| 7.750 | 7.750 | 7.750 | 6.833 | 6.833 |
| 7.917 | 7.917 | 7.917 | 6.833 | 7.833 |
| 7.417 | 7.417 | 7.417 | 6.833 | 6.833 |
| 6.417 | 6.417 | 6.417 | 6.833 | 6.833 |
| 8.000 | 8.333 | 8.167 | 6.833 | 7.833 |
| 5.750 | 5.583 | 5.667 | 4.167 | 4.167 |
| 7.917 | 7.917 | 7.917 | 7.833 | 7.833 |
| 5.083 | 5.083 | 5.083 | 5.000 | 5.000 |
| 7.917 | 7.917 | 7.917 | 7.833 | 7.833 |
| 7.333 | 7.333 | 7.333 | 5.750 | 6.833 |
| 7.750 | 7.917 | 7.834 | 6.833 | 6.833 |
| 7.250 | 6.750 | 7.000 | 6.833 | 6.833 |
| 7.917 | 8.083 | 8.000 | 7.833 | 6.833 |
| 5.750 | 5.750 | 5.750 | 5.750 | 5.750 |
| 6.833 | 6.833 | 6.833 | 5.750 | 5.750 |
| 7.333 | 7.333 | 7.333 | 6.833 | 6.833 |
| 6.250 | 5.750 | 6.000 | 5.000 | 5.750 |
| 5.833 | 5.500 | 5.667 | 5.750 | 5.750 |
| 8.750 | 9.250 | 9.000 | 8.833 | 8.833 |
| 7.083 | 7.083 | 7.083 | 6.833 | 6.833 |
| 7.583 | 7.583 | 7.583 | 6.833 | 6.833 |
| 9.167 | 9.167 | 9.167 | 8.833 | 8.833 |
| 4.750 | 4.750 | 4.750 | 1.500 | 3.000 |
| 4.583 | 4.750 | 4.667 | 1.500 | 3.000 |
| 4.917 | 4.917 | 4.917 | 2.000 | 2.750 |
| 5.083 | 5.083 | 5.083 | 4.000 | 4.000 |
| 5.167 | 5.167 | 5.167 | 1.500 | 2.750 |
| 4.667 | 4.667 | 4.667 | 3.500 | 4.000 |
| 4.250 | 4.250 | 4.250 | 4.000 | 3.500 |
| 5.000 | 5.167 | 5.084 | 4.000 | 4.000 |
| 5.333 | 5.333 | 5.333 | 2.000 | 3.000 |
| 5.167 | 5.167 | 5.167 | 4.500 | 5.000 |
| 4.833 | 4.833 | 4.833 | 4.000 | 3.500 |
| 5.083 | 5.250 | 5.167 | 4.000 | 3.500 |
| 5.333 | 5.333 | 5.333 | 3.500 | 4.000 |
| 5.167 | 5.167 | 5.167 | 3.500 | 4.000 |
| 4.833 | 4.833 | 4.833 | 4.000 | 4.500 |
| 5.000 | 4.833 | 4.917 | 4.000 | 4.500 |
| 4.250 | 4.250 | 4.250 | 3.500 | 3.500 |
| 4.083 | 4.083 | 4.083 | 4.000 | 4.500 |
| 4.000 | 4.500 | 4.250 | 4.000 | 4.000 |
| 2.583 | 2.750 | 2.667 | 2.000 | 2.750 |
| 4.500 | 4.667 | 4.584 | 4.500 | 4.500 |
| 4.000 | 4.333 | 4.167 | 3.500 | 3.500 |
| 5.500 | 5.500 | 5.500 | 3.500 | 4.000 |
| 4.500 | 4.500 | 4.500 | 5.000 | 5.000 |

| | | | | |
|---|---|---|---|---|
| 5.333 | 5.333 | 5.333 | 3.000 | 3.000 |
| 7.333 | 7.333 | 7.333 | 4.500 | 5.000 |
| 4.167 | 4.333 | 4.250 | 3.000 | 3.000 |
| 4.250 | 4.250 | 4.250 | 3.500 | 3.500 |
| 4.417 | 4.417 | 4.417 | 3.000 | 3.000 |
| 5.417 | 5.583 | 5.500 | 3.500 | 3.500 |
| 4.917 | 4.917 | 4.917 | 3.000 | 3.000 |
| 4.500 | 4.500 | 4.500 | 2.000 | 3.000 |
| 4.500 | 4.500 | 4.500 | 5.000 | 5.000 |
| 3.833 | 3.833 | 3.833 | 3.500 | 4.000 |
| 5.500 | 5.333 | 5.417 | 4.000 | 4.500 |
| 6.750 | 6.750 | 6.750 | 5.000 | 5.000 |
| 5.250 | 5.250 | 5.250 | 3.500 | 4.000 |
| 5.083 | 5.083 | 5.083 | 3.000 | 2.000 |
| 5.000 | 5.167 | 5.084 | 4.000 | 4.000 |
| 4.917 | 4.917 | 4.917 | 3.000 | 3.000 |
| 5.083 | 5.083 | 5.083 | 2.750 | 3.000 |
| 5.000 | 5.000 | 5.000 | 4.000 | 4.000 |
| 7.000 | 7.333 | 7.167 | 4.500 | 6.000 |
| 3.667 | 3.667 | 3.667 | 3.000 | 3.000 |
| 4.583 | 4.583 | 4.583 | 3.500 | 3.500 |
| 5.000 | 5.000 | 5.000 | 4.000 | 4.000 |
| 4.833 | 5.000 | 4.917 | 4.000 | 4.000 |
| 6.083 | 6.083 | 6.083 | 3.500 | 3.500 |
| 4.583 | 4.583 | 4.583 | 5.000 | 5.000 |
| 5.000 | 4.833 | 4.917 | 6.000 | 6.000 |
| 5.333 | 5.333 | 5.333 | 4.000 | 4.000 |
| 5.417 | 5.417 | 5.417 | 3.000 | 3.000 |
| 5.000 | 5.000 | 5.000 | 4.500 | 4.500 |
| 5.167 | 5.500 | 5.334 | 5.000 | 6.000 |
| 5.583 | 5.750 | 5.667 | 3.500 | 3.500 |
| 6.667 | 6.667 | 6.667 | 5.000 | 5.000 |
| 5.333 | 5.333 | 5.333 | 3.000 | 3.000 |
| 5.333 | 5.333 | 5.333 | 3.500 | 4.000 |
| 6.083 | 6.250 | 6.167 | 5.000 | 4.500 |
| 6.167 | 6.167 | 6.167 | 4.500 | 4.500 |
| 6.000 | 5.833 | 5.917 | 4.000 | 4.500 |
| 5.333 | 5.333 | 5.333 | 4.000 | 4.500 |
| 5.167 | 5.333 | 5.250 | 3.000 | 3.000 |
| 5.667 | 5.667 | 5.667 | 4.000 | 4.000 |
| 6.250 | 6.417 | 6.334 | 4.000 | 4.000 |
| 5.083 | 5.083 | 5.083 | 3.500 | 4.000 |
| 6.667 | 6.833 | 6.750 | 4.500 | 4.500 |
| 5.750 | 5.750 | 5.750 | 3.000 | 3.500 |
| 5.250 | 5.250 | 5.250 | 3.500 | 3.500 |
| 5.500 | 5.667 | 5.584 | 2.750 | 2.750 |
| 5.333 | 5.333 | 5.333 | 5.000 | 5.000 |
| 6.083 | 6.083 | 6.083 | 4.500 | 4.500 |
| 6.833 | 6.833 | 6.833 | 3.500 | 4.000 |
| 8.000 | 7.833 | 7.917 | 5.000 | 6.000 |
| 5.500 | 5.500 | 5.500 | 4.500 | 5.000 |
| 5.500 | 5.500 | 5.500 | 3.500 | 3.000 |
| 6.500 | 6.667 | 6.584 | 4.500 | 4.500 |
| 5.750 | 5.750 | 5.750 | 4.000 | 4.000 |
| 6.250 | 6.250 | 6.250 | 4.000 | 4.500 |
| 5.917 | 5.917 | 5.917 | 4.500 | 4.500 |
| 6.083 | 6.083 | 6.083 | 4.000 | 4.500 |
| 6.083 | 6.083 | 6.083 | 4.500 | 4.500 |

| | | | | |
|---|---|---|---|---|
| 5.417 | 5.417 | 5.417 | 4.500 | 4.500 |
| 6.500 | 7.000 | 6.750 | 5.000 | 5.000 |
| 5.917 | 5.917 | 5.917 | 4.500 | 4.500 |
| 6.167 | 6.167 | 6.167 | 4.500 | 4.500 |
| 6.667 | 6.667 | 6.667 | 6.000 | 6.000 |
| 7.000 | 7.000 | 7.000 | 5.000 | 5.000 |
| 7.000 | 7.167 | 7.084 | 4.500 | 5.000 |
| 6.333 | 6.333 | 6.333 | 5.000 | 5.000 |
| 6.250 | 6.250 | 6.250 | 4.500 | 4.500 |
| 6.750 | 6.750 | 6.750 | 3.500 | 4.000 |
| 5.667 | 5.833 | 5.750 | 4.500 | 4.500 |
| 6.417 | 6.417 | 6.417 | 5.000 | 5.000 |
| 7.333 | 7.333 | 7.333 | 4.000 | 4.500 |
| 6.750 | 6.750 | 6.750 | 5.000 | 5.000 |
| 6.250 | 6.417 | 6.334 | 4.000 | 4.000 |
| 6.417 | 6.417 | 6.417 | 4.000 | 4.500 |
| 7.750 | 7.750 | 7.750 | 6.000 | 6.000 |
| 6.833 | 6.833 | 6.833 | 4.000 | 4.000 |
| 6.417 | 6.583 | 6.500 | 4.500 | 4.500 |
| 6.500 | 6.500 | 6.500 | 5.000 | 5.000 |
| 6.500 | 6.500 | 6.500 | 5.000 | 5.000 |
| 6.500 | 6.667 | 6.584 | 5.000 | 5.000 |
| 6.417 | 6.417 | 6.417 | 4.000 | 4.000 |
| 6.667 | 6.667 | 6.667 | 4.000 | 4.000 |
| 6.500 | 6.333 | 6.417 | 4.000 | 4.500 |
| 6.750 | 6.750 | 6.750 | 4.500 | 4.500 |
| 6.500 | 6.500 | 6.500 | 4.500 | 4.500 |
| 6.000 | 6.167 | 6.084 | 4.500 | 5.000 |
| 7.333 | 7.333 | 7.333 | 5.000 | 6.000 |
| 6.833 | 6.833 | 6.833 | 5.000 | 5.000 |
| 5.333 | 5.333 | 5.333 | 3.500 | 3.500 |
| 7.500 | 7.167 | 7.334 | 5.000 | 5.000 |
| 7.083 | 6.917 | 7.000 | 4.500 | 4.500 |
| 6.833 | 6.833 | 6.833 | 4.000 | 4.500 |
| 8.167 | 8.167 | 8.167 | 6.000 | 6.000 |
| 6.917 | 7.083 | 7.000 | 5.000 | 5.000 |
| 6.500 | 6.500 | 6.500 | 4.500 | 5.000 |
| 6.833 | 6.833 | 6.833 | 4.000 | 4.500 |
| 6.833 | 6.833 | 6.833 | 5.000 | 5.000 |
| 6.500 | 7.000 | 6.750 | 4.500 | 5.000 |
| 7.000 | 7.000 | 7.000 | 6.000 | 6.000 |
| 7.167 | 7.167 | 7.167 | 4.500 | 5.000 |
| 7.083 | 7.250 | 7.167 | 6.000 | 6.000 |
| 7.167 | 7.167 | 7.167 | 6.000 | 6.000 |
| 6.500 | 6.500 | 6.500 | 5.000 | 5.000 |
| 7.833 | 7.500 | 7.667 | 4.500 | 5.000 |
| 7.167 | 7.167 | 7.167 | 6.000 | 6.000 |
| 8.167 | 8.167 | 8.167 | 4.000 | 4.000 |
| 7.417 | 7.417 | 7.417 | 4.500 | 4.500 |
| 7.417 | 7.250 | 7.334 | 5.000 | 5.000 |
| 5.500 | 5.500 | 5.500 | 4.000 | 4.000 |
| 8.083 | 8.083 | 8.083 | 6.000 | 6.000 |
| 7.083 | 6.917 | 7.000 | 6.000 | 6.000 |
| 6.500 | 6.667 | 6.584 | 5.000 | 5.000 |
| 6.083 | 6.250 | 6.167 | 4.500 | 4.500 |
| 6.000 | 6.000 | 6.000 | 5.000 | 5.000 |
| 7.083 | 7.083 | 7.083 | 5.000 | 5.000 |
| 7.750 | 7.750 | 7.750 | 4.500 | 4.500 |

| | | | | |
|---|---|---|---|---|
| 7.083 | 7.250 | 7.167 | 4.500 | 4.500 |
| 7.083 | 7.250 | 7.167 | 5.000 | 5.000 |
| 7.333 | 7.333 | 7.333 | 6.000 | 6.000 |
| 7.000 | 7.000 | 7.000 | 5.000 | 5.000 |
| 7.333 | 7.333 | 7.333 | 6.000 | 6.000 |
| 7.500 | 7.667 | 7.584 | 7.000 | 7.000 |
| 8.583 | 8.583 | 8.583 | 5.000 | 6.000 |
| 6.583 | 6.583 | 6.583 | 4.000 | 4.500 |
| 5.750 | 5.750 | 5.750 | 4.500 | 4.500 |
| 6.917 | 6.750 | 6.834 | 4.500 | 5.000 |
| 7.333 | 7.333 | 7.333 | 4.500 | 6.000 |
| 6.333 | 6.333 | 6.333 | 5.000 | 5.000 |
| 6.250 | 6.250 | 6.250 | 3.500 | 4.000 |
| 8.083 | 7.917 | 8.000 | 5.000 | 6.000 |
| 7.167 | 7.167 | 7.167 | 7.000 | 7.000 |
| 7.000 | 7.000 | 7.000 | 4.500 | 5.000 |
| 8.417 | 8.417 | 8.417 | 6.000 | 6.000 |
| 7.500 | 7.667 | 7.584 | 6.000 | 6.000 |
| 7.167 | 7.167 | 7.167 | 5.000 | 5.000 |
| 7.000 | 7.000 | 7.000 | 5.000 | 5.000 |
| 6.833 | 6.833 | 6.833 | 5.000 | 6.000 |
| 7.833 | 7.667 | 7.750 | 6.000 | 7.000 |
| 8.000 | 8.167 | 8.084 | 6.000 | 6.000 |
| 6.667 | 6.667 | 6.667 | 5.000 | 5.000 |
| 7.000 | 7.000 | 7.000 | 5.000 | 5.000 |
| 6.333 | 6.333 | 6.333 | 5.000 | 6.000 |
| 7.250 | 7.417 | 7.334 | 6.000 | 6.000 |
| 6.417 | 6.417 | 6.417 | 4.000 | 4.000 |
| 7.333 | 7.333 | 7.333 | 6.000 | 6.000 |
| 6.917 | 7.083 | 7.000 | 5.000 | 6.000 |
| 7.583 | 7.583 | 7.583 | 6.000 | 6.000 |
| 7.583 | 7.583 | 7.583 | 5.000 | 5.000 |
| 7.417 | 7.583 | 7.500 | 6.000 | 6.000 |
| 7.583 | 7.583 | 7.583 | 6.000 | 6.000 |
| 7.583 | 7.583 | 7.583 | 6.000 | 6.000 |
| 7.583 | 7.583 | 7.583 | 6.000 | 7.000 |
| 6.583 | 6.750 | 6.667 | 4.000 | 4.000 |
| 7.083 | 7.250 | 7.167 | 6.000 | 5.000 |
| 6.000 | 6.000 | 6.000 | 4.500 | 4.500 |
| 6.667 | 6.667 | 6.667 | 6.000 | 6.000 |
| 5.750 | 5.917 | 5.834 | 4.000 | 4.500 |
| 7.917 | 7.917 | 7.917 | 5.000 | 6.000 |
| 6.833 | 6.833 | 6.833 | 5.000 | 6.000 |

| average_GP | D1_CA-TW3 | D2_CA-RUS-CHN | D3_CA-GP |
|---|---|---|---|
| 3.500 | -1.667 | -1.917 | -0.500 |
| 3.500 | -1.083 | -2.083 | -0.500 |
| 3.834 | -1.167 | -1.917 | -0.834 |
| 3.250 | -1.417 | -1.584 | -0.167 |
| 2.250 | -0.917 | -1.751 | 0.833 |
| 3.500 | -1.000 | -0.750 | -0.333 |
| 4.167 | -1.583 | -1.750 | -0.917 |
| 2.500 | -1.167 | -1.584 | 0.833 |
| 3.500 | -0.167 | -1.584 | -0.167 |
| 4.167 | -1.083 | -1.666 | -0.750 |
| 3.000 | -0.666 | -1.500 | 0.417 |
| 3.500 | -0.250 | -1.833 | 0.000 |
| 3.000 | -0.333 | -1.750 | 0.500 |
| 4.167 | -0.583 | -1.250 | -0.667 |
| 4.167 | -2.000 | -0.084 | -0.584 |
| 4.167 | -0.333 | -2.167 | -0.500 |
| 3.834 | -1.416 | 0.167 | -0.167 |
| 3.500 | -0.500 | -1.500 | 0.167 |
| 5.000 | -0.333 | -1.417 | -1.250 |
| 3.500 | -0.417 | -1.667 | 0.333 |
| 4.167 | -1.250 | -1.000 | -0.334 |
| 3.834 | -1.166 | -0.250 | 0.083 |
| 4.250 | -0.583 | -0.166 | -0.333 |
| 5.000 | -1.333 | -0.666 | -1.083 |
| 4.167 | -1.250 | -1.417 | -0.167 |
| 5.750 | -1.750 | -2.250 | -1.667 |
| 5.000 | -0.917 | -1.500 | -0.917 |
| 4.584 | -0.750 | -1.250 | -0.501 |
| 4.625 | -0.917 | -1.167 | -0.542 |
| 3.000 | -0.583 | -0.750 | 1.167 |
| 4.584 | -0.833 | -1.000 | -0.334 |
| 5.000 | -0.833 | -1.000 | -0.750 |
| 5.375 | -1.250 | -1.834 | -1.125 |
| 4.167 | -0.333 | -0.750 | 0.083 |
| 4.375 | -0.167 | -0.917 | -0.125 |
| 5.375 | -1.750 | -2.500 | -1.125 |
| 3.250 | 0.333 | 0.250 | 1.083 |
| 4.584 | -0.583 | -1.083 | -0.167 |
| 4.959 | -0.417 | -0.500 | -0.459 |
| 3.834 | 0.417 | -1.000 | 0.667 |
| 4.584 | -0.583 | -0.917 | -0.083 |
| 4.917 | -0.417 | -0.501 | -0.334 |
| 3.500 | -0.917 | -0.334 | 1.083 |
| 4.167 | -0.084 | -0.667 | 0.416 |
| 5.750 | -1.833 | -2.083 | -1.083 |
| 4.584 | -0.333 | -0.916 | 0.083 |
| 5.000 | -1.083 | -1.583 | -0.333 |
| 4.625 | -0.666 | -1.250 | 0.042 |
| 4.584 | -0.667 | -0.750 | 0.167 |
| 5.375 | -1.000 | -1.334 | -0.625 |
| 5.750 | -1.000 | -1.417 | -1.000 |
| 4.584 | -0.167 | -0.250 | 0.167 |
| 4.584 | -0.500 | -0.667 | 0.167 |
| 4.167 | -0.333 | -0.750 | 0.583 |
| 5.500 | -0.917 | -1.584 | -0.667 |
| 5.375 | -0.750 | -2.000 | -0.542 |
| 3.834 | -0.666 | -0.750 | 1.084 |

| | | | |
|---|---|---|---|
| 4.584 | -1.666 | -2.166 | 0.334 |
| 3.500 | -1.250 | -1.500 | 1.417 |
| 3.000 | 1.417 | -0.917 | 1.917 |
| 5.000 | -0.333 | -0.417 | 0.000 |
| 5.750 | -0.583 | -0.667 | -0.750 |
| 3.500 | 0.417 | 0.333 | 1.500 |
| 3.250 | 0.417 | 0.250 | 1.750 |
| 3.500 | -0.083 | -0.583 | 1.500 |
| 5.000 | -0.417 | -0.417 | 0.083 |
| 4.167 | -0.500 | -0.584 | 0.916 |
| 4.584 | -0.500 | -0.584 | 0.500 |
| 4.584 | -0.417 | -0.584 | 0.500 |
| 3.500 | 0.916 | 0.583 | 1.583 |
| 3.834 | 0.250 | -0.584 | 1.250 |
| 5.000 | -0.500 | -0.584 | 0.083 |
| 3.834 | -0.417 | -0.584 | 1.250 |
| 4.167 | -0.416 | -0.500 | 1.000 |
| 4.167 | -0.166 | -0.500 | 1.000 |
| 4.167 | -0.166 | -0.250 | 1.000 |
| 4.167 | -0.500 | -0.250 | 1.083 |
| 4.167 | -0.500 | -0.625 | 1.083 |
| 5.000 | -0.500 | -0.583 | 0.250 |
| 3.834 | 0.250 | 0.083 | 1.417 |
| 4.955 | -0.667 | -0.667 | 0.378 |
| 5.000 | -0.666 | -0.833 | 0.417 |
| 5.375 | -0.916 | -1.250 | 0.042 |
| 4.584 | -0.583 | -0.167 | 0.834 |
| 4.167 | -0.166 | -0.250 | 1.250 |
| 5.000 | -0.083 | -0.083 | 0.417 |
| 6.292 | -1.250 | -1.333 | -0.875 |
| 4.584 | -0.166 | -0.250 | 0.834 |
| 4.167 | -0.083 | -0.166 | 1.250 |
| 5.000 | -0.167 | -0.333 | 0.500 |
| 5.750 | -0.833 | -0.917 | -0.250 |
| 5.000 | -0.583 | -0.708 | 0.500 |
| 6.292 | -0.583 | -1.167 | -0.792 |
| 5.750 | -1.083 | -2.417 | -0.250 |
| 4.167 | 0.000 | -0.083 | 1.416 |
| 6.833 | -1.417 | -2.001 | -1.250 |
| 5.000 | -0.417 | -0.584 | 0.583 |
| 6.292 | -1.000 | -1.500 | -0.709 |
| 5.375 | -0.500 | -1.833 | 0.292 |
| 5.000 | -0.083 | -0.333 | 0.667 |
| 5.750 | -0.083 | -0.333 | -0.083 |
| 5.750 | -0.083 | -0.166 | -0.083 |
| 5.750 | -0.083 | -0.166 | -0.083 |
| 6.833 | -1.333 | -2.000 | -1.166 |
| 5.750 | -0.250 | -0.500 | 0.000 |
| 4.167 | -0.333 | -0.417 | 1.583 |
| 5.000 | 0.167 | 0.083 | 0.750 |
| 5.375 | -0.333 | -0.417 | 0.375 |
| 5.000 | -0.250 | -0.250 | 0.750 |
| 5.000 | 0.333 | 0.000 | 0.833 |
| 5.000 | -0.250 | -0.334 | 0.833 |
| 4.584 | -0.917 | 0.000 | 1.250 |
| 5.750 | -0.417 | -0.750 | 0.083 |
| 5.750 | -0.250 | -0.584 | 0.083 |
| 5.000 | 0.333 | 0.250 | 0.833 |

| | | | |
|---|---|---|---|
| 4.167 | 0.000 | -0.167 | 1.666 |
| 5.375 | -0.417 | -0.751 | 0.458 |
| 6.292 | -0.417 | -0.917 | -0.459 |
| 5.750 | -0.083 | -0.250 | 0.167 |
| 6.292 | -0.916 | -1.000 | -0.375 |
| 5.750 | -0.166 | -0.250 | 0.167 |
| 5.750 | -0.250 | -0.833 | 0.167 |
| 5.000 | -0.083 | -0.583 | 0.917 |
| 5.500 | -0.250 | -0.250 | 0.417 |
| 5.750 | -0.250 | -0.583 | 0.167 |
| 5.375 | -0.583 | -0.583 | 0.542 |
| 3.834 | 0.834 | -0.417 | 2.084 |
| 5.750 | 0.000 | -0.167 | 0.250 |
| 5.000 | -0.167 | -0.167 | 1.000 |
| 5.375 | 0.167 | 0.000 | 0.625 |
| 5.750 | -0.250 | -0.417 | 0.250 |
| 5.000 | 0.417 | 0.333 | 1.000 |
| 5.750 | -1.000 | -1.083 | 0.250 |
| 3.250 | 1.000 | 1.000 | 2.750 |
| 5.750 | -0.250 | -0.667 | 0.250 |
| 8.833 | -3.167 | -3.500 | -2.833 |
| 6.292 | -1.000 | -1.500 | -0.209 |
| 5.917 | -0.417 | -0.917 | 0.167 |
| 6.292 | -0.584 | -1.417 | -0.209 |
| 5.750 | 0.250 | 0.083 | 0.333 |
| 6.292 | 0.083 | 0.083 | -0.209 |
| 5.750 | -2.584 | -2.584 | 0.333 |
| 6.292 | -1.417 | -1.750 | -0.209 |
| 5.750 | -0.250 | -0.583 | 0.417 |
| 5.750 | 0.417 | 0.167 | 0.417 |
| 4.584 | 0.584 | 0.500 | 1.584 |
| 4.584 | 0.084 | 0.000 | 1.584 |
| 7.333 | -1.583 | -2.250 | -1.166 |
| 4.167 | 0.917 | 0.917 | 2.000 |
| 5.750 | 0.167 | -0.250 | 0.417 |
| 4.167 | 0.667 | 0.500 | 2.000 |
| 6.833 | -0.333 | -1.000 | -0.666 |
| 6.833 | -0.250 | -1.083 | -0.583 |
| 3.500 | 1.250 | 1.084 | 2.750 |
| 6.833 | -1.583 | -1.750 | -0.583 |
| 4.167 | 1.250 | 1.084 | 2.083 |
| 5.375 | 0.750 | 0.417 | 0.875 |
| 5.750 | 0.500 | 0.417 | 0.500 |
| 3.500 | 1.167 | 1.084 | 2.750 |
| 6.292 | -0.917 | -1.500 | -0.042 |
| 6.833 | -1.750 | -2.167 | -0.583 |
| 5.750 | 0.750 | 0.666 | 0.583 |
| 5.000 | 1.000 | 0.916 | 1.333 |
| 5.750 | 0.583 | 0.333 | 0.583 |
| 6.292 | -0.583 | -0.583 | 0.126 |
| 6.833 | -0.833 | -0.917 | -0.416 |
| 6.833 | -0.666 | -0.667 | -0.416 |
| 5.750 | -0.583 | -1.333 | 0.667 |
| 5.833 | -0.416 | -0.083 | 0.584 |
| 6.833 | -0.833 | -0.833 | -0.416 |
| 5.750 | 0.834 | 0.750 | 0.667 |
| 5.000 | 0.250 | 0.167 | 1.417 |
| 5.000 | 0.417 | 0.250 | 1.417 |

| | | | |
|---|---|---|---|
| 5.375 | -0.083 | -0.083 | 1.042 |
| 5.000 | 1.417 | 0.834 | 1.417 |
| 6.792 | -0.500 | -0.583 | -0.292 |
| 6.292 | -0.583 | -0.833 | 0.209 |
| 6.833 | -1.083 | -1.333 | -0.333 |
| 7.333 | -0.667 | -0.667 | -0.750 |
| 5.000 | 0.833 | 0.750 | 1.583 |
| 3.834 | 1.000 | 0.917 | 2.750 |
| 7.333 | -0.917 | -1.334 | -0.750 |
| 4.584 | 0.916 | 0.916 | 2.000 |
| 7.833 | -1.583 | -2.083 | -1.166 |
| 7.333 | -0.833 | -1.333 | -0.666 |
| 6.833 | -0.500 | -1.083 | -0.166 |
| 7.333 | -0.833 | -1.250 | -0.666 |
| 6.833 | -0.166 | -0.750 | -0.166 |
| 6.833 | 0.417 | 0.250 | -0.166 |
| 7.333 | -0.500 | -1.417 | -0.583 |
| 4.167 | 1.167 | 1.084 | 2.583 |
| 7.833 | -0.917 | -1.167 | -1.083 |
| 5.000 | 1.750 | 1.667 | 1.750 |
| 7.833 | -0.917 | -1.167 | -1.083 |
| 6.292 | -0.334 | -0.500 | 0.542 |
| 6.833 | -0.417 | -1.001 | 0.000 |
| 6.833 | -0.667 | -0.167 | 0.000 |
| 7.333 | -0.167 | -1.167 | -0.500 |
| 5.750 | 1.333 | 1.083 | 1.083 |
| 5.750 | 0.500 | 0.000 | 1.083 |
| 6.833 | -0.083 | -0.416 | 0.084 |
| 5.375 | 1.084 | 0.917 | 1.542 |
| 5.750 | 1.417 | 1.251 | 1.167 |
| 8.833 | -1.666 | -2.083 | -1.916 |
| 6.833 | -0.166 | -0.166 | 0.084 |
| 6.833 | -0.083 | -0.666 | 0.084 |
| 8.833 | -2.583 | -2.250 | -1.916 |
| 2.250 | -0.25 | -1.75 | 0.750 |
| 2.250 | -0.167 | -1.5835 | 0.833 |
| 2.375 | -1.417 | -1.834 | 0.708 |
| 4.000 | 0 | -1.833 | -0.750 |
| 2.125 | -0.8335 | -1.917 | 1.125 |
| 3.750 | 0.1665 | -1.417 | -0.500 |
| 3.750 | -1.5835 | -0.917 | -0.417 |
| 4.000 | -0.5835 | -1.7505 | -0.667 |
| 2.500 | 0.3335 | -1.916 | 0.917 |
| 4.750 | -0.583 | -1.75 | -1.333 |
| 3.750 | -1.75 | -1.333 | -0.250 |
| 3.750 | -0.833 | -1.6665 | -0.250 |
| 3.750 | -0.5005 | -1.75 | -0.167 |
| 3.750 | -1.25 | -1.5 | -0.083 |
| 4.250 | -1.4165 | -1.166 | -0.583 |
| 4.250 | -1 | -1.2495 | -0.583 |
| 3.500 | -0.833 | -0.583 | 0.167 |
| 4.250 | -1.417 | -0.333 | -0.500 |
| 4.000 | -0.5835 | -0.5 | -0.250 |
| 2.375 | 0.6665 | 1.1665 | 1.458 |
| 4.500 | -0.416 | -0.6665 | -0.583 |
| 3.500 | -1.416 | -0.2495 | 0.417 |
| 3.750 | -0.833 | -1.583 | 0.167 |
| 5.000 | -0.666 | -0.583 | -1.083 |

| | | | |
|---|---|---|---|
| 3.000 | -0.75 | -1.333 | 1.000 |
| 4.750 | -2.667 | -3.333 | -0.750 |
| 3.000 | 0 | -0.25 | 1.000 |
| 3.500 | -0.0835 | -0.25 | 0.500 |
| 3.000 | -0.25 | -0.417 | 1.000 |
| 3.500 | -1.167 | -1.5 | 0.500 |
| 3.000 | -0.417 | -0.834 | 1.083 |
| 2.500 | -0.084 | -0.417 | 1.583 |
| 5.000 | -0.084 | -0.417 | -0.917 |
| 3.750 | 0.084 | 0.334 | 0.417 |
| 4.250 | -0.75 | -1.1665 | 0.000 |
| 5.000 | -2.25 | -2.5 | -0.750 |
| 3.750 | -0.6665 | -1 | 0.500 |
| 2.500 | -0.5 | -0.833 | 1.750 |
| 4.000 | -0.5 | -0.8335 | 0.250 |
| 3.000 | -0.25 | -0.667 | 1.250 |
| 2.875 | -0.5 | -0.833 | 1.375 |
| 4.000 | -0.667 | -0.75 | 0.250 |
| 5.250 | -2.342 | -2.8335 | -0.917 |
| 3.000 | 0.417 | 0.75 | 1.417 |
| 3.500 | 0.25 | -0.166 | 0.917 |
| 4.000 | -0.333 | -0.5 | 0.500 |
| 4.000 | -0.083 | -0.4165 | 0.500 |
| 3.500 | -0.5 | -1.583 | 1.000 |
| 5.000 | -0.833 | -0.083 | -0.500 |
| 6.000 | -0.167 | -0.4165 | -1.500 |
| 4.000 | 0 | -0.75 | 0.583 |
| 3.000 | -0.2505 | -0.834 | 1.583 |
| 4.500 | 0.083 | -0.417 | 0.083 |
| 5.500 | -1.5005 | -0.7505 | -0.917 |
| 3.500 | 0.33385 | -0.9995 | 1.167 |
| 5.000 | -1.833 | -2 | -0.333 |
| 3.000 | -0.2495 | -0.666 | 1.667 |
| 3.750 | -0.25 | -0.666 | 0.917 |
| 4.750 | -1.2495 | -1.4995 | -0.083 |
| 4.500 | -1.25 | -1.5 | 0.167 |
| 4.250 | -1.083 | -1.1665 | 0.500 |
| 4.250 | -0.167 | -0.5 | 0.583 |
| 3.000 | -0.167 | -0.417 | 1.833 |
| 4.000 | -0.417 | -0.834 | 0.833 |
| 4.000 | -1.25 | -1.5005 | 0.833 |
| 3.750 | 0.334 | -0.166 | 1.167 |
| 4.500 | -1.333 | -1.833 | 0.417 |
| 3.250 | -0.333 | -0.833 | 1.667 |
| 3.500 | 0.084 | -0.333 | 1.417 |
| 2.750 | -0.083 | -0.6665 | 2.167 |
| 5.000 | 0 | -0.333 | 0.000 |
| 4.500 | -0.5 | -1.083 | 0.500 |
| 3.750 | -0.917 | -1.75 | 1.333 |
| 5.500 | -2.5 | -2.8335 | -0.417 |
| 4.750 | -0.25 | -0.417 | 0.333 |
| 3.250 | 0 | -0.417 | 1.833 |
| 4.500 | -1.083 | -1.4165 | 0.667 |
| 4.000 | 0.167 | -0.583 | 1.167 |
| 4.250 | -0.583 | -1.083 | 0.917 |
| 4.500 | -0.083 | -0.667 | 0.750 |
| 4.250 | -0.25 | -0.833 | 1.000 |
| 4.500 | -0.25 | -0.833 | 0.750 |

| | | | |
|---|---|---|---|
| 4.500 | 0 | -0.084 | 0.833 |
| 5.000 | -0.417 | -1.417 | 0.333 |
| 4.500 | -0.167 | -0.584 | 0.833 |
| 4.500 | -0.166 | -0.75 | 0.917 |
| 6.000 | -0.666 | -1.25 | -0.583 |
| 5.000 | -1.250 | -1.583 | 0.417 |
| 4.750 | -0.833 | -1.667 | 0.667 |
| 5.000 | -0.416 | -0.916 | 0.417 |
| 4.500 | -0.250 | -0.750 | 1.000 |
| 3.750 | -0.833 | -1.250 | 1.750 |
| 4.500 | 0.333 | -0.167 | 1.083 |
| 5.000 | -0.166 | -0.750 | 0.667 |
| 4.250 | -0.916 | -1.666 | 1.417 |
| 5.000 | -0.500 | -1.000 | 0.750 |
| 4.000 | 0.000 | -0.584 | 1.750 |
| 4.250 | -0.083 | -0.667 | 1.500 |
| 6.000 | -1.334 | -1.917 | -0.167 |
| 4.000 | -0.417 | -1.000 | 1.833 |
| 4.500 | -0.251 | -0.667 | 1.333 |
| 5.000 | -0.167 | -0.667 | 0.833 |
| 5.000 | -0.167 | -0.667 | 0.833 |
| 5.000 | -0.334 | -0.751 | 0.833 |
| 4.000 | -0.167 | -0.584 | 1.833 |
| 4.000 | -0.417 | -0.834 | 1.833 |
| 4.250 | -0.083 | -0.500 | 1.667 |
| 4.500 | -0.416 | -0.833 | 1.417 |
| 4.500 | -0.250 | -0.583 | 1.417 |
| 4.750 | -0.250 | -0.083 | 1.250 |
| 5.500 | -1.000 | -1.333 | 0.500 |
| 5.000 | -0.333 | -0.833 | 1.000 |
| 3.500 | 1.000 | 0.667 | 2.500 |
| 5.000 | -0.667 | -1.334 | 1.000 |
| 4.500 | -0.667 | -1.000 | 1.500 |
| 4.250 | -0.417 | -0.750 | 1.833 |
| 6.000 | -1.750 | -2.084 | 0.083 |
| 5.000 | -0.584 | -0.917 | 1.083 |
| 4.750 | 0.250 | -0.417 | 1.333 |
| 4.250 | -0.417 | -0.750 | 1.833 |
| 5.000 | -0.250 | -0.750 | 1.083 |
| 4.750 | -0.250 | -0.667 | 1.333 |
| 6.000 | -0.500 | -0.833 | 0.167 |
| 4.750 | -0.583 | -1.000 | 1.417 |
| 6.000 | -0.500 | -1.000 | 0.167 |
| 6.000 | -0.583 | -1.000 | 0.167 |
| 5.000 | 0.167 | -0.333 | 1.167 |
| 4.750 | -0.917 | -1.500 | 1.417 |
| 6.000 | -0.500 | -0.917 | 0.250 |
| 4.000 | -1.584 | -1.917 | 2.250 |
| 4.500 | -0.500 | -1.167 | 1.750 |
| 5.000 | -0.833 | -1.084 | 1.250 |
| 4.000 | 1.167 | 0.750 | 2.250 |
| 6.000 | -1.333 | -1.833 | 0.250 |
| 6.000 | -0.334 | -0.667 | 0.333 |
| 5.000 | 0.166 | -0.251 | 1.333 |
| 4.500 | 0.583 | 0.167 | 1.833 |
| 5.000 | 0.666 | 0.333 | 1.333 |
| 5.000 | -0.334 | -0.750 | 1.333 |
| 4.500 | -0.750 | -1.333 | 1.917 |

| | | | |
|---|---|---|---|
| 4.500 | -0.333 | -0.750 | 1.917 |
| 5.000 | -0.416 | -0.750 | 1.417 |
| 6.000 | -0.666 | -0.916 | 0.417 |
| 5.000 | -0.250 | -0.583 | 1.417 |
| 6.000 | -0.500 | -0.833 | 0.500 |
| 7.000 | -0.667 | -1.084 | -0.500 |
| 5.500 | -1.834 | -2.083 | 1.000 |
| 4.250 | 0.500 | -0.083 | 2.250 |
| 4.500 | 1.250 | 0.750 | 2.000 |
| 4.750 | 0.250 | -0.251 | 1.833 |
| 5.250 | -0.501 | -0.750 | 1.333 |
| 5.000 | 0.750 | 0.250 | 1.583 |
| 3.750 | 1.000 | 0.417 | 2.917 |
| 5.500 | -0.916 | -1.333 | 1.167 |
| 7.000 | -0.083 | -0.500 | -0.333 |
| 4.750 | 0.000 | -0.333 | 1.917 |
| 6.000 | -1.166 | -1.750 | 0.667 |
| 6.000 | -0.500 | -0.917 | 0.667 |
| 5.000 | -0.083 | -0.500 | 1.667 |
| 5.000 | 0.417 | -0.333 | 1.667 |
| 5.500 | 0.417 | -0.083 | 1.250 |
| 6.500 | -0.583 | -1.000 | 0.250 |
| 6.000 | -1.000 | -1.334 | 0.750 |
| 5.000 | 0.500 | 0.083 | 1.750 |
| 5.000 | 0.083 | -0.250 | 1.750 |
| 5.500 | 1.000 | 0.417 | 1.250 |
| 6.000 | -0.333 | -0.584 | 0.750 |
| 4.000 | 0.917 | 0.333 | 2.750 |
| 6.000 | -0.251 | -0.500 | 0.833 |
| 5.500 | 0.166 | -0.167 | 1.333 |
| 6.000 | -0.334 | -0.750 | 0.833 |
| 5.000 | -0.334 | -0.750 | 1.833 |
| 6.000 | -0.250 | -0.667 | 0.833 |
| 6.000 | -0.334 | -0.750 | 0.833 |
| 6.000 | -0.334 | -0.750 | 0.833 |
| 6.500 | -0.334 | -0.750 | 0.333 |
| 4.000 | 0.667 | 0.251 | 2.917 |
| 5.500 | 0.167 | -0.250 | 1.417 |
| 4.500 | 1.417 | 0.917 | 2.417 |
| 6.000 | 0.667 | 0.250 | 0.917 |
| 4.250 | 1.584 | 1.084 | 2.667 |
| 5.500 | -0.583 | -1.000 | 1.417 |
| 5.500 | 0.417 | 0.084 | 1.417 |
